# Multidisciplinary study of the secondary immune response in grandparents re-exposed to chickenpox

**DOI:** 10.1038/s41598-017-01024-8

**Published:** 2017-04-24

**Authors:** B. Ogunjimi, J. Van den Bergh, P. Meysman, S. Heynderickx, K. Bergs, H. Jansens, E. Leuridan, A. Vorsters, H. Goossens, K. Laukens, N. Cools, Viggo Van Tendeloo, N. Hens, P. Van Damme, Evelien Smits, Ph. Beutels

**Affiliations:** 1 Centre for Health Economics Research & Modeling Infectious Diseases (CHERMID), Vaccine & Infectious Disease Institute (VAXINFECTIO), University of Antwerp, Antwerp, Belgium; 2 Interuniversity Institute for Biostatistics and Statistical Bioinformatics, Hasselt University, Hasselt, Belgium; 3 Department of Pediatrics, Antwerp University Hospital, Edegem, Belgium; 4 Laboratory of Experimental Hematology (LEH), Vaccine & Infectious Disease Institute (VAXINFECTIO), University of Antwerp, Antwerp, Belgium; 5 Department of Pediatrics, Ghent University Hospital, Ghent, Belgium; 6 Antwerp Unit for Data Analysis and Computation in Immunology and Sequencing (AUDACIS), Antwerp, Belgium; 7 Advanced Database Research and Modelling (ADReM), Department of Mathematics and Computer Science, University of Antwerp, Antwerp, Belgium; 8 Biomedical Informatics Research Network Antwerp (BIOMINA), University of Antwerp/Antwerp University Hospital, Edegem, Belgium; 9 Center for Cell Therapy and Regenerative Medicine, Antwerp University Hospital, Edegem, Belgium; 10 Department of Laboratory Medicine, Antwerp University Hospital, Edegem, Belgium; 11 Centre for the Evaluation of Vaccination (CEV), Vaccine & Infectious Disease Institute (VAXINFECTIO), University of Antwerp, Antwerp, Belgium; 12 Laboratory of Medical Microbiology, Vaccine & Infectious Disease Institute (VAXINFECTIO), University of Antwerp, Antwerp, Belgium; 13 Center for Oncological Research Antwerp, University of Antwerp, Antwerp, Belgium; 14 School of Public Health and Community Medicine, The University of New South Wales, Sydney, Australia

## Abstract

Re-exposure to chickenpox may boost varicella-zoster virus (VZV) immunity in the elderly. This secondary immune response is hypothesized to confer protection against herpes zoster. We longitudinally sampled 36 adults over the course of one year after re-exposure to chickenpox. The resulting 183 samples and those of 14 controls were assessed for VZV-specific T-cell immunity and antibody titres. The percentages of VZV-specific CD4+ IL-2-producing T-cells were increased in re-exposed grandparents compared to control participants up to 9 months after re-exposure. Using a longitudinal mixture modelling approach, we found that 25% and 17% of re-exposed grandparents showed a boosting of VZV-specific CD4+ IL-2-producing T-cells and VZV-specific antibodies, respectively. The antibody boosting occurred exclusively in cytomegalovirus (CMV) IgG-positive participants. CMV IgG-positive participants also had higher VZV IE62-specific CD4+ IFN-γ-producing T-cell percentages and VZV-specific antibody titres. The protective effect of re-exposure to chickenpox is likely limited, as boosting only occurred in 17–25% of the VZV re-exposed grandparents and for less than one year.

## Introduction

Many studies have focused on the characterization of the immune response in an experimental design (i.e., in an animal model, after vaccination or after another artificially induced challenge). However, the study of secondary immune responses in humans after a natural re-exposure has received far less attention. A notable exception is a series of studies that focused on immune responses following the re-exposure to varicella-zoster virus (VZV)^1–4^. As such, the analysis of the dynamics of the VZV-specific immune response after re-exposure to chickenpox can offer important insights into the fundamentals of the secondary immune response after real life re-exposure.

Furthermore, given that re-exposure to VZV is assumed to boost VZV cellular immunity (referred to as “exogenous boosting”) and that herpes zoster, the (symptomatic) reactivation of VZV that had previously remained latent in neural ganglia after chickenpox, is likely to be caused by a reduced level of VZV-specific cellular immunity, re-exposure to VZV was hypothesized to reduce the risk of herpes zoster^5^. This hypothesis has had an important influence on policy making concerning universal childhood VZV vaccination. Simulation models exploring this hypothesis concluded that diminished circulating chickenpox, after the introduction of universal chickenpox vaccination, would cause a temporary increase in herpes zoster incidence^5–9^. These simulation results are largely driven by the more intensive intergenerational contacts between children and their parents and grandparents and an assumed direct inverse proportionality between the number of contacts with chickenpox cases and the probability of developing herpes zoster. Since the disease burden - expressed as Quality Adjusted Life Years (QALYs) losses - is typically weighted 10 to 20 times more for an average herpes zoster case than for an average chickenpox case, the overall public health impact of universal childhood VZV vaccination produced by such simulations tends to be negative^10^. Observational data in countries with universal childhood VZV vaccination so far cannot convincingly reject the occurrence of such an undesirable population impact, leading to continuing hesitance towards introducing universal VZV vaccination in many countries worldwide.

Therefore, both for biomedical insights and public health, it is important to adequately assess the VZV-specific immune response following a re-exposure to chickenpox. Until now, only a few studies have investigated the VZV-specific immune response following re-exposure to varicella, and these have focused almost exclusively on re-exposed parents^1,2,4^. The bulk of the burden of herpes zoster is, however, carried by older adults^11^. The boosting studies in young adults showed a boosting of the cellular immune response in 60–70% of participants, although the quantification was limited. In the current study, we set out to analyse the characteristics of the secondary immune response in grandparents, starting shortly after they contacted their grandchild experiencing chickenpox.

## Methods

### Participants

Thirty-six grandparents (median age 59 years, range 47–70; 24/36 women) were recruited after being re-exposed to chickenpox for a minimum of four hours and within five days of varicella exanthema eruption in their grandchildren. These chickenpox patients were either determined to be VZV PCR-positive by skin or saliva swabs (21/25 children) and/or clinically diagnosed by a medical doctor. The re-exposed grandparents were longitudinally sampled beginning “as soon as possible after re-exposure” and at 3 weeks, 6 weeks, 15 weeks, 30 weeks and up to 52 weeks after re-exposure. Fourteen individuals (median age 58 years, range 48–68; 10/14 women) without known re-exposures to chickenpox during the last year were selected as controls. Nine of the 14 controls were longitudinally sampled up to 52 weeks after the first sample was taken. Five controls contributed a single blood sample.

This study was approved by the ethics board of the Antwerp University Hospital. All methods were performed in accordance with the relevant guidelines and regulations when applicable. Written informed consent was obtained from all study participants.

### Blood processing and/or cytometric analyses

Peripheral blood mononuclear cells (PBMCs) were isolated by Ficoll-Paque Plus gradient separation (Amersham Biosciences, Uppsala, Sweden) from freshly obtained heparinized blood. The PBMCs were frozen in 90% foetal bovine serum (FBS; Life Technologies, Ghent, Belgium) supplemented with 10% dimethyl sulphoxide (DMSO; Sigma-Aldrich, Steinheim, Germany). Filled cryovials were placed in Nalgene cryoboxes before freezing at −80 °C, and after 24 hours, the cells were stored in liquid nitrogen. The thawing of the cells for batch analysis was performed in a water bath at 37 °C. After washing with pre-heated (37 °C) medium supplemented with 10% FBS, a flow cytometric analysis was performed. An intermittent batch-wise analysis was performed in order to minimize inter-assay variability.

PBMCs were either unstimulated or stimulated overnight with phytohaemagglutinin (PHA; 2 µg/mL) + ionomycin (1 µg/mL), VZV IE62 PepMix, VZV IE63 PepMix or VZV gE PepMix (all peptide mixes were from JPT and consisted of 15-mer peptides with an 11-aa overlap, Berlin, Germany). The overnight stimulation was performed in the presence of GolgiPlug (BD). Afterwards, the cells were washed and stained using the antibody panel described in Table 1. The cells were first stained with antibodies that bind membrane markers. Then, the cells were fixed and permeabilized using Fix&Lyse buffer and Perm 2 solution (BD) according to the manufacturer’s protocol before staining for intracellular cytokines, i.e., IFN-γ and IL-2.

**Table 1:** Antibody panel for PBMC staining.

Target	Fluorochrome	Clone	Company
IFN-γ	FITC	B27	BD
CCR7	PE	150503	BD
CD3	PE-TexasRed	S4.1	Life Technologies
CD45RA	PE-Cy7	HI100	BD
CD28	PE-Cy5 or PerCP-Cy5.5	CD28.2 L293	BD
Live/Dead	Violet		Life Technologies
IL-2	APC	5344.111	BD
CD8	Pacific Orange	3B5	Life Technologies
CD4	APC-H7	RPA-T4	BD

The CD28-specific antibody was changed after approximately one year of study duration. Five control participants and ten re-exposed grandparents had week 30–52 samples that were analysed using both the new and the old antibody panel. After stimulation with VZV IE62, VZV IE63 or VZV gE, we detected no statistically significant differences between the two panels with a different choice of anti-CD28 antibody.

Flow cytometric analyses were performed on a BDFACS Aria II flow cytometer (BD Biosciences, Erembodegem, Belgium). FlowJo software (Tree Star Inc., Ashland, OR) was used for data analysis.

### VZV-specific CD8+ T-cell receptor sequencing

PBMCs from three re-exposed grandparents, collected approximately 15 weeks after re-exposure, were thawed and recovered overnight in Iscove’s Modified Dulbecco’s Medium (IMDM; Invitrogen, Merelbeke, Belgium). One half of the PBMCs was used in a 1-day-culture protocol, while the other half was used in a 1-week-culture protocol. In both protocols, the PBMCs were stimulated with 20 µg/mL Varicella Zoster grade 2 Antigen (VZV lysate) (Microbiox, Ontario, Canada) at day 0 and incubated for 24 hours at 37 °C, 3.5% O_2_ and 5% CO_2_. For the 1-week-culture protocol, 30 ng/mL interleukin (IL)-21 (at day 0) and 12.5 U/mL IL-2, 5 ng/mL IL-7 and 5 ng/mL IL-15 (at day +2) (Immunotools, Friesoythe, Germany) were added to maintain the culture. At day +6, the culture was restimulated by adding 20 µg/mL VZV lysate. For both protocols, CD8^+^ T cells were isolated from the (re)stimulated PBMCs by positive selection using magnetic CD8 microbeads (Miltenyi Biotec, Utrecht, The Netherlands) according to the manufacturer’s instructions. In a second step, CD8+ CD137+ T cells were further isolated using a flow cytometric sorting process. Briefly, the isolated CD8^+^ T cells were stained with anti-CD3, anti-CD8, and anti-CD137 antibodies and a Live/Dead stain, followed by a sorting of the living CD3^+^CD8^+^CD137^+^ T cells with a FACSAria II instrument (BD, Erembodegem, Belgium). Total DNA from the isolated cells was obtained using a QIAamp DNA Micro Kit (Qiagen, Hilden, Germany) according to the manufacturer’s instructions. The eluates of the 1-day and 1-week cultures were pooled and frozen at −20 °C until further processing. The T-cell receptor beta (TCRβ) sequencing on the DNA was performed by Adaptive Biotechnologies (Seattle, USA).

### Whole blood T-cell receptor sequencing

DNA from the three grandparents was extracted from 200,000 CD8+ T-cells per subject at three different time points, without prior selection after stimulation, as detailed above for the VZV-specific TCR sequencing. The TCRβ sequencing was performed by Adaptive Biotechnologies (Seattle, USA).

### Antibody titration

Serum was stored at −80 °C until further processing. VZV-specific IgG antibody titres (mIU/ml) in thawed serum were determined using VZV-infected cell lysate on a Liaison XL instrument with a DiaSorin kit (Italy)^3^. The presence of IgGs directed against CMV pp150, pp28, p38, and p52 in thawed serum was determined using a Roche Elecsys assay (Basel, Switzerland).

### Data management

The data management and analyses were performed using SPSS v20 and Monolix v4.3.3. The results were termed “significant” if the p-value was < 0.05.

Per individual, sample and stimulation condition, the net percentages of IFN-γ or IL-2-producing CD3+CD4+ T-cells and CD3+CD8+ T-cells were calculated using Fisher’s Exact test (see ref.^3^). Due to the scarcity of IL-2-producing CD8+ T-cells, these data were not shown in the paper. In addition, we used a data processing algorithm per setting (individual, stimulus, cytokine, CD4 or CD8 T-cell) that excluded cytokine-producing T-cell percentages if (1) the stimulus did not evoke a significant cytokine response AND the count of parent cells was lower than 10000 or if (2) the stimulus did not evoke a significant cytokine response AND the background cytokine response (the cytokine response in a sample without stimulus) was higher than 0.15%. We used basic nonparametric statistics (Mann-Whitney U test given the non-normal distribution of the data and limited sample size or Fisher’s Exact Test for contingency table analyses) to compare the immune responses between the different time points for the re-exposed grandparents and the median immune response for each control. Based on a qualitative assessment of previously published results^4,12,13^, the different time points were categorized as ≤1 week, >1 week and ≤4 weeks, >4 weeks and ≤7 weeks, >7 weeks and ≤20 weeks, >20 weeks and ≤39 weeks, and >39 weeks. Each time category thus contained the results from samples obtained within the defined time period corresponding to the category. Not all individuals could be sampled in each time category, so the number of included data points varies between time points. To formally identify different subsets of individuals, we also applied a mixture modelling approach that was based on the longitudinal dynamics of the VZV-specific immune response. This could be modelled using a system of differential equations (in the case of antibodies) or damped exponential equations (in the case of T-cells). To achieve this, we developed a longitudinal mixed (regression) model. These are mathematical models that include parameters with fixed and random components. Time is a continuous variable in these models. The fixed component of a parameter can be interpreted as a conditional mean value for the parameter. The random component, however, represents an individual-specific deviation from the conditional mean value. This means that heterogeneity can be modelled using random effects. In our models, the random parameters were assumed to follow a lognormal distribution. In addition to the longitudinal mixed modelling, we also used a between-subjects mixture modelling approach, as available in Monolix 4.3.3 (Lixoft, France)^14^. Here, we assume that the immune response (e.g., antibody titre) of each individual can be described by different equations with (fixed + random) parameters. In the mixture modelling approach, the statistical algorithm will estimate for each individual which equation is most adequate in describing the individual-specific immune response at all time points. The longitudinal data analyses were performed using Monolix 4.3.3 software. The parameter estimates were obtained using the Maximum Likelihood Estimation by the Stochastic Approximation Expectation Maximization (SAEM) algorithm. Stochastic approximation was used to calculate the Fisher Information Matrix and subsequently the standard errors per parameter. Importance sampling was used to determine the log-likelihood per model. The models were compared using Akaike’s Information Criterion (AIC, averaged over three runs per model)^15^. In the first step, the application of random effects was assessed in a forward regression algorithm, where we started from no random effects (thus only fixed effects) and subsequently computed the AIC value after adding a random effect to each of the parameters separately. In a second step, we started from the model with the best AIC for the fixed + random effect for a certain parameter and computed the AIC values again when adding random effects to the other parameters that yielded convergence in the first step (and so on). For several models, likelihood computation was not possible (no MCMC convergence or ill-defined model).

## Results

### VZV-specific antibody titres from grandparents re-exposed to chickenpox do not differ between different time points after re-exposure

**Figure 1:**
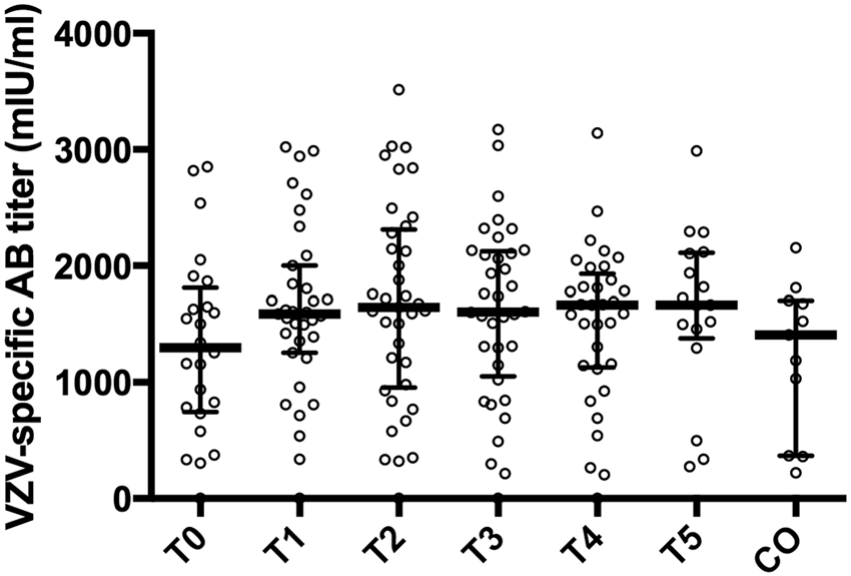
VZV-specific antibody titres. VZV-specific antibody titres are shown, using median and interquartile range, up to one year after re-exposure to chickenpox (T0, ≤1 week; T1, >1 week and ≤4 weeks; T2, >4 weeks and ≤7 weeks; T3, >7 weeks and ≤20 weeks; T4, >20 weeks and ≤39 weeks; T5, >39 weeks) and in control participants (CO). The numbers of samples per sample group are 24 (T0), 35 (T1), 37 (T2), 36 (T3), 33 (T4), 17 (T5) and 14 (CO). No significant differences were found.

Thirty-six grandparents donated blood samples for up to one year after re-exposure to chickenpox. The VZV-specific antibody titres did not differ significantly between different time points after re-exposure (see Supplementary Table S1 and Fig. 1). Moreover, these VZV-specific antibody titres did not differ from the VZV-specific antibody titres from 14 control participants (see Supplementary Table S1 and Fig. 1). Interestingly, one out of the 36 grandparents showed no VZV-specific antibody response at any of the time points. This grandparent was a recently retired paediatric nurse who likely has had many exposures to chickenpox during her professional life.

### Re-exposed grandparents only exhibit a short-term increase in VZV-specific CD4+ IL-2-producing T-cells compared to controls

**Figure 2:**
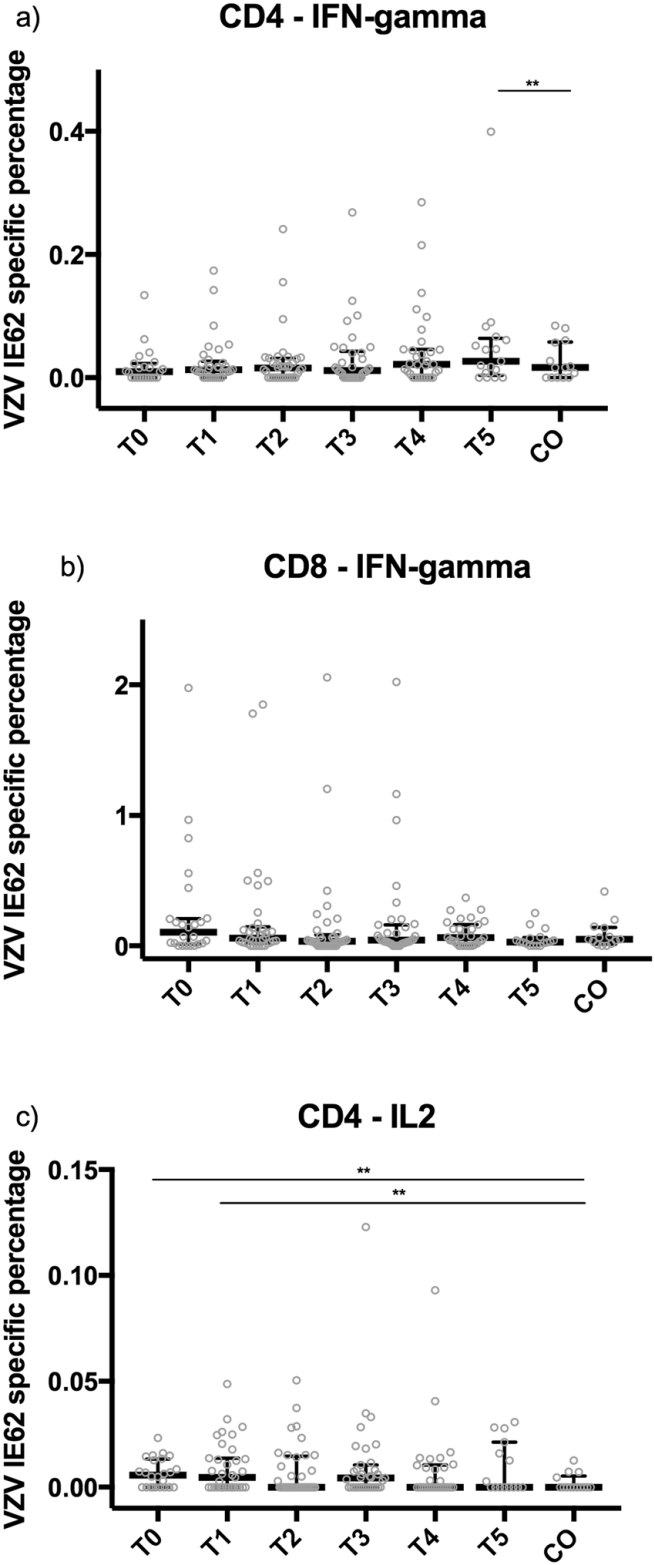
T-cell responses against VZV IE62. Percentages of CD4+ and CD8+ IFN-γ-producing and CD4+ IL-2-producing T-cells against VZV IE62 are shown, using median and interquartile range, up to one year after re-exposure to chickenpox (T0, ≤1 week; T1, >1 week and ≤4 weeks; T2, >4 weeks and ≤7 weeks; T3, >7 weeks and ≤20 weeks; T4, >20 weeks and ≤39 weeks; T5, >39 weeks) and in control participants (CO). The number of samples per sample group are: for IFN-γ-producing T-cells, 23 (T0), 34 (T1), 36 (T2), 34 (T3), 32 (T4), 17 (T5), 14 (CO); for CD4+ IL-2-producing T-cells, 24 (T0), 34 (T1), 37 (T2), 35 (T3), 32 (T4), 17 (T5), 14 (CO); and for CD8+ IFN-γ-producing T-cells, 24 (T0), 35 (T1), 37 (T2), 35 (T3), 32 (T4), 15 (T5), 14 (CO). **p-value < 0.05.

**Figure 3:**
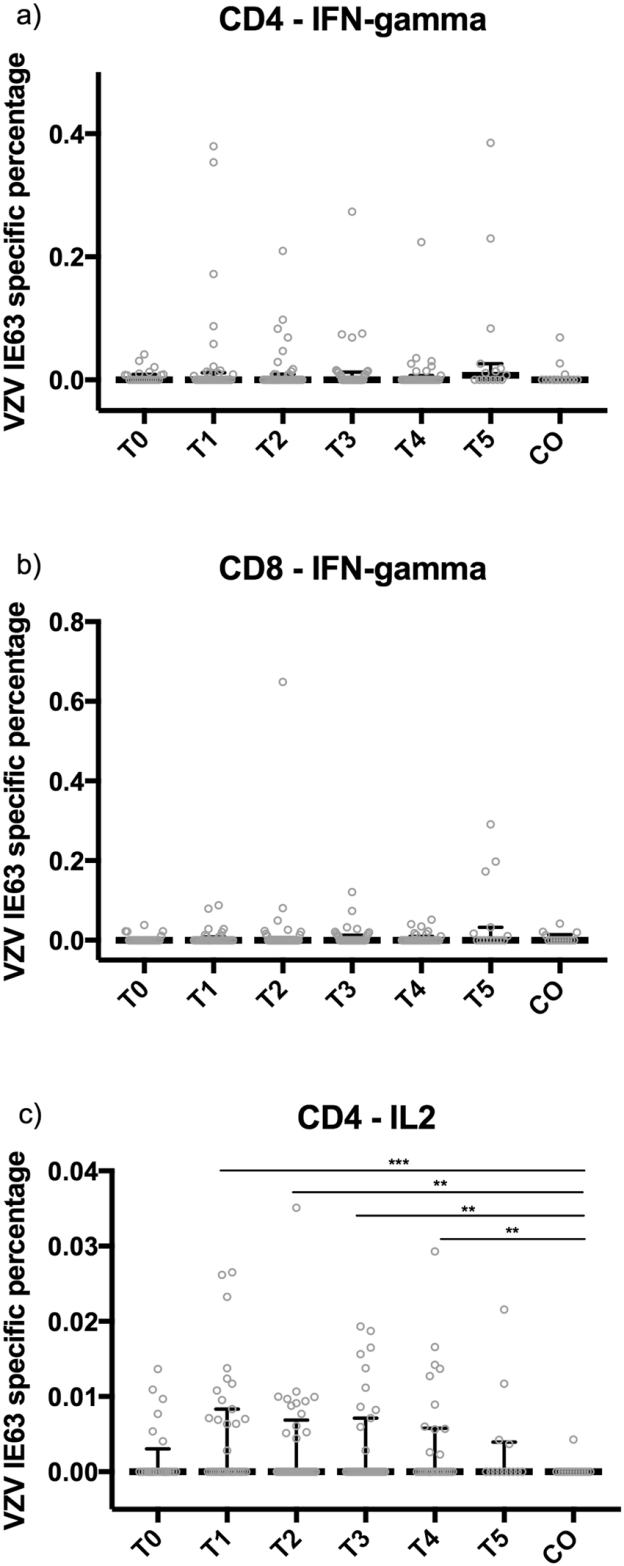
T-cell responses against VZV IE63. Percentages of CD4+ and CD8+ IFN-γ-producing and CD4+ IL-2-producing T-cells against VZV IE63 are shown, using median and interquartile range, up to one year after re-exposure to chickenpox (T0, ≤1 week; T1, >1 week and ≤4 weeks; T2, >4 weeks and ≤7 weeks; T3, >7 weeks and ≤20 weeks; T4, >20 weeks and ≤39 weeks; T5, >39 weeks) and in control participants (CO). The number of samples per sample group are: for IFN-γ-producing T-cells, 23 (T0), 34 (T1), 37 (T2), 33 (T3), 31 (T4), 15 (T5), 14 (CO); for CD4+ IL-2-producing T-cells, 24 (T0), 35 (T1), 37 (T2), 36 (T3), 31 (T4), 15 (T5), 14 (CO); and for CD8+ IFN-γ-producing T-cells, 24 (T0), 35 (T1), 37 (T2), 36 (T3), 30 (T4), 13 (T5), 14 (CO). **p-value < 0.05; ***p-value < 0.01.

**Figure 4:**
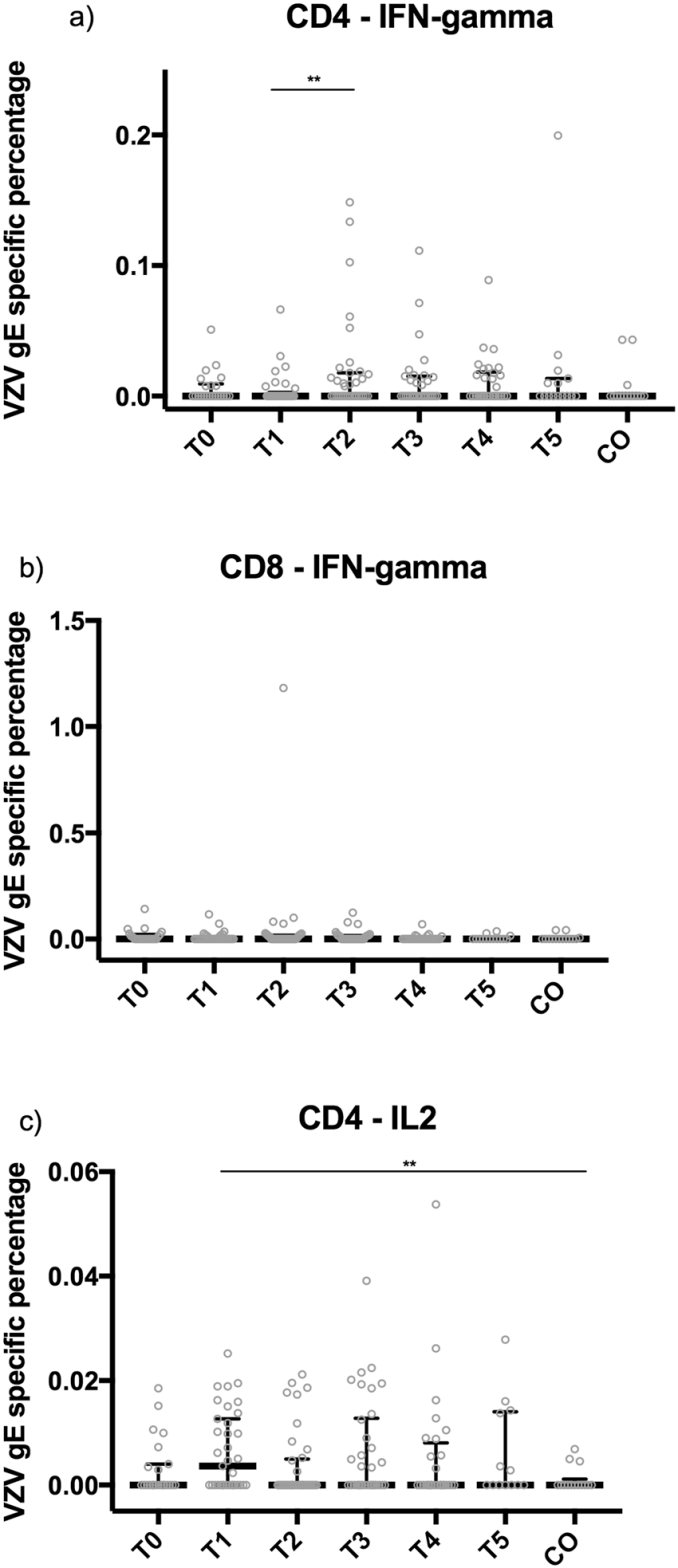
T-cell responses against VZV gE. Percentages of CD4+ and CD8+ IFN-γ-producing and CD4+ IL-2-producing T-cells against VZV gE are shown, using median and interquartile range, up to one year after re-exposure to chickenpox (T0, ≤1 week; T1, >1 week and ≤4 weeks; T2, >4 weeks and ≤7 weeks; T3, >7 weeks and ≤20 weeks; T4, >20 weeks and ≤39 weeks; T5, >39 weeks) and in control participants (CO). The number of samples per sample group are: for IFN-γ-producing T-cells, 23 (T0), 33 (T1), 36 (T2), 33 (T3), 30 (T4), 15 (T5), 12 (CO); for CD4+ IL-2-producing T-cells, 23 (T0), 35 (T1), 37 (T2), 34 (T3), 29 (T4), 14 (T5), 12 (CO); and for CD8+ IFN-γ-producing T-cells, 23 (T0), 35 (T1), 37 (T2), 34 (T3), 28 (T4), 13 (T5), 12 (CO). **p-value < 0.05.

CD4+ and CD8+ T-cells were stimulated with VZV IE62, VZV IE63 and VZV gE peptide mixes, and the percentages of IFN-γ-producing and IL-2-producing CD4+ or CD8+ T-cells were compared between different time points as well as with those of control participants. No systematic significant differences in the percentages of CD4+ or CD8+ IFN-γ-producing T-cells generated by VZV IE62, VZV IE63 and VZV gE stimulation were detected between the samples from the grandparents at different time points or compared to those from control participants (see Supplementary Table S1 and Figs 2 and 3). Although no systematic and significant differences were detected for CD4+ IL-2-producing T-cells against any of the VZV peptides between different time points for the re-exposed grandparents, CD4+ IL-2-producing T-cells were significantly more prevalent in re-exposed grandparents versus the control participants at all time points between weeks 1 and 39 for VZV IE63, up to 4 weeks for VZV IE62 and between 1 and 4 weeks after re-exposure for VZV gE (see Supplementary Table S1 and Figs 2 and 4).

### VZV-specific T-cells show no change in phenotype after re-exposure to chickenpox

The CD4+ and CD8+ T-cells were further subtyped using CCR7 and CD45RA into effector-memory (CCR7−CD45RA−), central memory (CCR7+CD45RA−), late effector (CCR7−CD45RA+) and naïve (CCR7+CD45RA+) T-cells. Next, we assessed (using the Kruskal-Wallis U test statistic) for each subtype and for each VZV peptide (IE62, IE63 and gE) whether a change was noted for the T-cells from individuals who showed cytokine production against the peptide (see Supplementary Figure 1). We only detected changes for VZV IE62 IL-2-producing CD4+ late effector T-cells (p-value 0.014). However, given the lack of systematic results, we may infer that no evidence for systematic T-cell phenotype dynamics was noted after re-exposure to chickenpox.

### Longitudinal TCRβ sequencing of VZV-specific CD8+ T-cells reveals no boosting in grandparents after re-exposure to chickenpox

PBMCs from three grandparents, each sampled at three different time points (5d/41d/207d, 2d/18d/199d, and 4d/39d/187d after re-exposure) were cultured using VZV-lysate. After one week, the CD137+CD8+ T-cells were sorted for DNA extraction. Next, TCR**β** sequencing was performed using these VZV-specific CD8+ T-cells (see Supplementary Figure 1). We focused on CD8+ T-cells because a recent study highlighted the importance of MHC I for prolonged herpes zoster^16^. For each of the three grandparents, the Spearman correlations between TCR**β** read counts from the three samples were highly correlated, with all correlations being higher than 0.9 with p-values < 0.0001. Signed rank comparisons for the TCR**β** read counts between the three time points per grandparent showed no significant differences for the grandparents.

### Mathematical longitudinal models indicate the boosting of VZV-specific antibodies in 1 out of 6 grandparents after re-exposure to chickenpox

We hypothesized that perhaps not all re-exposed grandparents were exogenously boosted and used between-subjects longitudinal mixture modelling to formally distinguish between boosted and non-boosted re-exposed grandparents. This mathematical method allows for an exploratory approach towards the statistical classification of longitudinal trajectories from individuals into several equations. Given the modest sample size and the larger experimental error for the T-cell data, we first focused on VZV-specific antibody titres and used the following between-subjects mixture model (modified version from Andraud *et al*.^17^):

1 $$\{\begin{array}{rcl}\frac{dA{B}_{1}}{dt} & = & pL+pS\cdot {S}_{0}\cdot {e}^{-uS\cdot t}-uAB\cdot AB\\ A{B}_{2} & = & iAB\end{array}$$

with AB_1_ and AB_2_ representing the post-re-exposure VZV-specific antibody titres for two different trajectories, pS the antibody production rate for short-living antibody secreting cells (SASC), S_0_ the initial SASC concentration, uS the SASC decay rate, pL the constant antibody production rate by long-living antibody-secreting cells (LASC), uAB the antibody decay rate, and iAB the initial antibody concentration (which is the constant value for equation AB_2_). The main goal of the mathematical models in this paper was not to arrive at an exact representation of the underlying immunological processes, but rather to produce adequate model fits to our immunological measurements. Six out of the 36 (17%) re-exposed grandparents were found to follow the boosting dynamics represented by the differential equation for AB_1_, whereas 30/36 grandparents were found to have a constant value represented by the equation for AB2 (see Supplementary Figure 3).

Next, we focused on longitudinal mixture modelling of VZV-specific cellular immunity. Here, due to higher experimental variability, we chose to work with more flexible curve fitting by means of a damped exponential equation model:

2 $$\{\begin{array}{rcl}{T}_{1} & = & (a\cdot t+b)\cdot {e}^{-c\cdot t}+d\\ {T}_{2} & = & e\end{array}$$

with T_1_ and T_2_ representing the number of cytokine-producing cells (distinct models for IFN-γ and IL-2) for two different types of trajectories, a, b, c and d parameters for the damped exponential function, and *e* a constant number of cytokine-producing cells for individuals following equation T2.

We chose to only model CD4+ IL-2+, CD4+ IFN-γ+ and CD8+ IFN-γ+ T-cells against VZV IE62 because of the higher prevalence of zero value results for the other conditions. Moreover, no MCMC convergence was obtained for the CD4+ IFN-γ-producing T-cell dataset. Although the modelling analysis for the CD8+ IFN-γ-producing T-cell data showed MCMC convergence, the goodness of fit was much better for the CD4+ IL-2-producing T-cell data against VZV IE62 (Supplementary Figure 4). The modelling for the CD4+ IL-2-producing VZV IE62-specific T-cells classified 9/36 (25%) individuals as boosted, whereas 27/36 were considered to have a constant value.

Interestingly, the individuals whose antibody titres were found to be boosted were not the same as those whose CD4+ IL-2-producing T-cell data were boosted.

### CMV seropositivity is associated with the boosting of VZV-specific antibodies and with an overall higher VZV-specific T-cell response

We assessed whether gender or CMV IgG seropositivity (22/36 were CMV-seropositive) had an effect on exogenous boosting. Using a contingency table approach (Fisher’s Exact test) on the VZV antibody and CD4+ IL-2 VZV IE62 T-cell data, we found that gender had no significant effect on boosting nor did CMV seropositivity on the CD4+ IL-2+ boosting for VZV IE62-specific T-cells. However, all six individuals who were determined to have been exogenously boosted using the longitudinal mixture modelling approach were CMV seropositive versus 16/30 that were not boosted (p-value 0.039).

**Figure 5:**
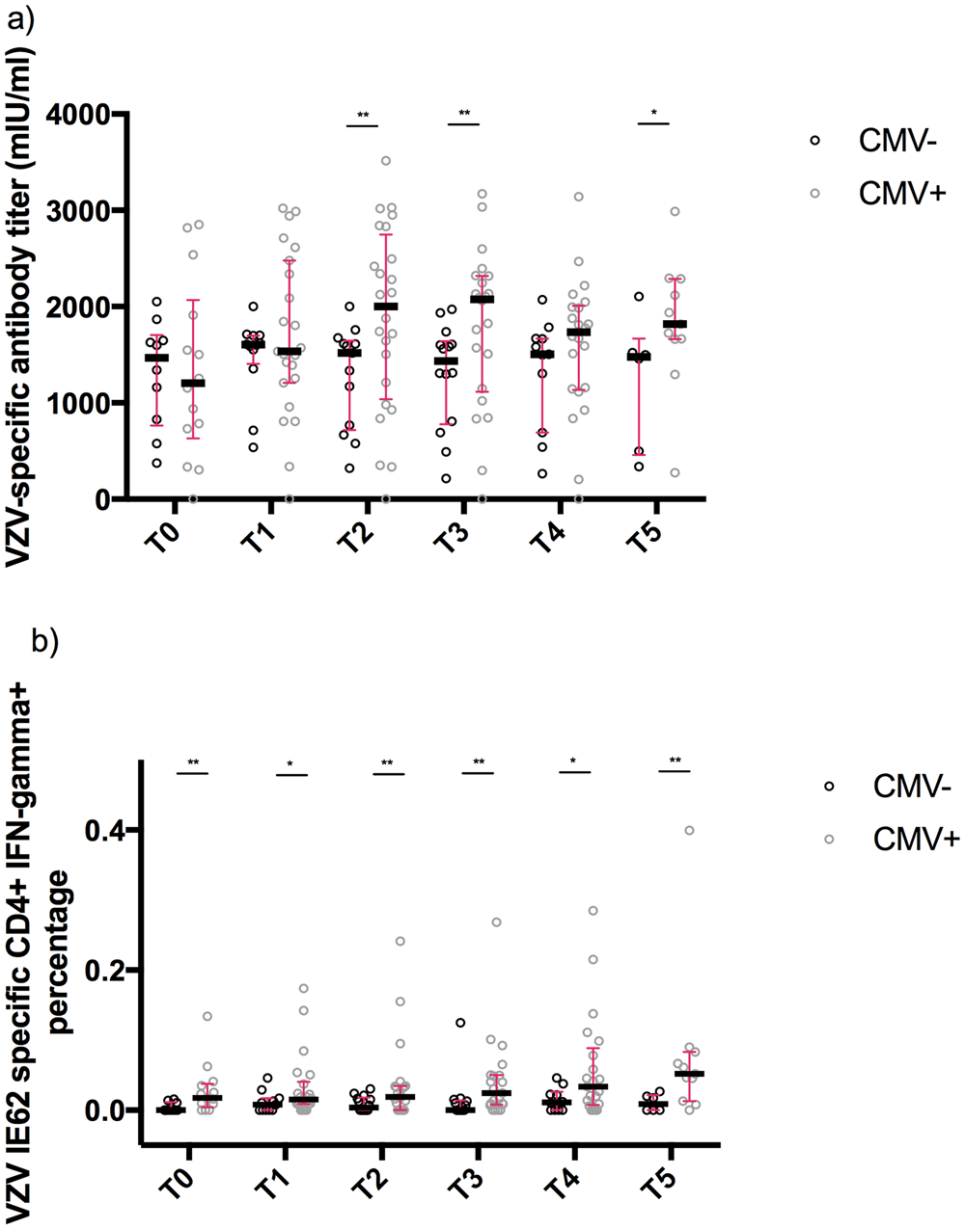
Effect of CMV seropositivity on VZV-specific immunity. (**a**) VZV-specific antibody titres are shown, using median and interquartile range, up to one year after re-exposure to chickenpox (T0, ≤1 week; T1, >1 week and ≤4 weeks; T2, >4 weeks and ≤7 weeks; T3, >7 weeks and ≤20 weeks; T4, >20 weeks and ≤39 weeks; T5, >39 weeks) as a function of CMV seropositivity. (**b**) Percentages of CD4+ IFN-γ-producing T-cells against VZV IE62 are shown, using median and interquartile range, up to one year after re-exposure to chickenpox (T0, ≤1 week; T1, >1 week and ≤4 weeks; T2, >4 weeks and ≤7 weeks; T3, >7 weeks and ≤20 weeks; T4, >20 weeks and ≤39 weeks; T5, >39 weeks) as a function of CMV seropositivity. *p-value < 0.1; **p-value < 0.05.

Next, we assessed whether CMV seropositivity had an effect on the VZV-specific immune response (see Supplementary Table S2). We found that CMV-seropositive grandparents had a higher VZV-specific antibody response at 2/6 time points (Fig. 5), whereas there was a clear overall tendency for a higher VZV IE62-specific CD4+ IFN-γ-producing T-cell percentage at all time points (Fig. 5). No systematic significant effects for CMV seropositivity on the other T-cell responses were noted nor were there any systematic significant effects noted for gender on VZV-specific immune responses.

## Discussion

In this pilot study, we used a multidisciplinary in-depth approach to study the secondary immune response in grandparents after re-exposure to chickenpox.

We found that VZV-specific antibody titres did not systematically show boosting after re-exposure, nor were they higher in re-exposed grandparents compared to control participants. Our findings were consistent with the finding that paediatricians have similar VZV-specific antibody titres as control adults^3^ and that no systematic signs of the boosting of VZV-specific antibodies were observed in re-exposed parents^1,2,4^.

Next, we showed that the percentages of CD4+ or CD8+ T-cells that produced IFN-γ after stimulation with VZV IE62, VZV IE63 or VZV gE did not significantly differ between different time points after re-exposure to chickenpox in grandparents, nor was there a significant difference with those observed in the control participants. We also did not detect a difference between time points for CD4+ IL-2-producing T-cells directed against the VZV peptides. Our study did show, however, that the re-exposed grandparents had higher percentages of CD4+ IL-2-producing T-cells compared to control participants for up to 40 weeks after re-exposure against the various VZV peptides. Possibly, the discrepancy between the lack of overall boosting of individual CD4+ IL-2-producing T-cell trajectories in grandparents and the higher CD4+ IL-2-producing T-cell response in grandparents compared to control participants can be explained by a rapid and steady elevation of these cells after re-exposure to VZV. We also used novel TCRβ sequencing technology for an exploratory in-depth assessment of the VZV-specific CD8+ T-cell response^18^. These results were consistent with our previous findings, as we found no proof of exogenous boosting in the re-exposed grandparents using TCRβ sequencing.

We hypothesized that perhaps not all grandparents were successfully boosted after re-exposure, and we used a longitudinal mixture modelling analysis to formally distinguish between boosted and non-boosted grandparents. This formal method allowed us to estimate that 17% of grandparents showed a boosting of the VZV-specific antibody titre, whereas 25% of grandparents showed a boosting of VZV IE62 specific CD4+ IL-2-producing T-cells.

The lack of a pronounced and systematic boosting of VZV-specific immunity after exogenous re-exposure to VZV is noteworthy, as many vaccine-based studies have shown robust boosting of both VZV-specific antibodies and T-cells after vaccination, even in older individuals^12,13,19,20^. This lack of a robust and systematic boosting in our study could be explained by the difference in antigen-presentation route. Indeed, saliva contains VZV-specific IgA that could be a barrier to the systematic distribution of VZV to lymph nodes (where subsequent activation and proliferation is expected)^21^. However, re-exposure to chickenpox has been shown to induce the boosting of VZV-specific T-cells in 60–70% of re-exposed parents^1,4^. The ad hoc approach towards defining “boosting” in those previous parent studies contrasts with our formal statistical approach to detect boosting events in the current study, and may have led to the finding of a relatively high proportion of boosted parents. In addition, it has been shown that the T-cell response after VZV vaccination decreases with age^13^. If this finding can be extrapolated to exogenous re-exposure, this could mean that grandparents are less susceptible to boosting than parents. The latter hypothesis is further supported by the recent suggestions that immunosenescence is not only caused by CMV-dependent mechanisms but CMV-independent mechanisms as well^22,23^.

Interestingly, our study showed that CMV-seropositive grandparents were more likely to show boosting of VZV-specific antibodies after re-exposure to chickenpox. Some (but not all) studies have shown an increased immune response after vaccination against influenza in CMV-seropositive individuals^23^. It has been argued that chronic CMV infection leads to an overall increased activation of the immune system^23^. Our study also showed overall higher (cellular and humoral) VZV-specific immune responses in CMV-seropositive grandparents. Together, our observations can be explained by a more activated and responsive immune system in CMV-seropositive adults. However, this does not align with our recent observation that CMV seropositivity is associated with an increased risk of HZ in adults younger than 50 years^24^. One alternative hypothesis could be that a chronic CMV infection, shown to have led to more differentiated and possibly less equipped T-cell immunity against various pathogens including VZV^25^, has weakened the immune barriers against VZV and thereby has led to a more pronounced *in vivo* spreading of VZV (and subsequent boosting). This hypothesis could explain why only CMV-seropositive grandparents showed a boosting of antibodies and why CMV-seropositive grandparents had higher cellular and humoral immune responses against VZV. Unfortunately, our study did not include a measurement of VZV viraemia. Interestingly, a recent serological study on VZV-specific antibody titres as a function of age also implied that chronic CMV infection could lead to more frequent VZV reactivation and subsequent boosting (termed endogenous boosting^22^). Both hypotheses (CMV as an activator of immunity versus CMV as a depressor of immunity) could explain the observations made in this study. More research is clearly needed to disentangle the effects of CMV and ageing on VZV dynamics.

The results from this study are of importance beyond the realm of fundamental immunology, as the exogenous boosting hypothesis is considered to be an obstacle for the implementation of universal chickenpox vaccination^6^. Many cost-effectiveness studies have concluded that the decrease of contacts with chickenpox patients after the implementation of universal chickenpox vaccination would lead to a significant increase of herpes zoster incidence, which could overshadow the positive effects on the reduction of chickenpox^7,8,26^. These simulation models were based on the deterministic modelling estimation that re-exposure to chickenpox would lead to a duration of protection against herpes zoster for 20 years^27^. However, a recent individual-based model for VZV estimated the duration of exogenous boosting to last for 1–2 years^9^. Our experimental study supports the latter modelling estimation through our findings that (average) boosting of cellular immunity was not noticeable by one year after re-exposure (vs. control participants). This means that our data suggest that the cost-effectiveness analyses for chickenpox vaccination should be redone using the new experimental results, potentially leading to a policy in favour of universal chickenpox vaccination.

Our pilot study encountered several limitations, including the modest sample size, the exclusive focus on grandparents and the exclusive use of peptide mixes for the stimulation of PBMCs. We note that differences in exposure characteristics might also have had an effect on boosting potential as more infectious children (more than 50 vesicles) have been estimated to have mildly increased infectivity^28^. However, the estimated number of vesicles (more or less than 50) was in our study not associated with the boosting of antibodies or CD4+ IL-2-producing T-cells (data not shown). In addition, the lack of “true” baseline samples could have led to an underestimation of boosting. Furthermore, more frequent sampling during the first weeks after re-exposure could also give more insights into boosting dynamics, as recent studies on T-cell dynamics and TCR dynamics following VZV vaccination highlighted a peak in VZV-specific T-cells about one week after vaccination^19,20^. Future larger studies should also encompass both parents and grandparents so that the effect of immunosenescence could receive more attention. These larger studies will be more suitable for the mathematical classification of boosted and non-boosted individuals. Indeed, we noted that the formal classification of boosting did not correlate with our visual classification in all instances. Moreover, it would be interesting if other cytokines besides IFN-γ and IL-2 could also be included in the assessment.

In conclusion, we have found empirical evidence for short-lasting boosting of the cellular immune response against VZV in a minority of grandparents after re-exposure to chickenpox. CMV-seropositive individuals were shown to have higher cellular and humoral immune responses against VZV than CMV-seronegative individuals. In addition, the VZV-specific humoral immune response was boosted only in CMV-seropositive grandparents.

## Electronic supplementary material

Supplementary Figures

Supplementary Table 1

Supplementary Table 2
