# Author Correction: Multidisciplinary study of the secondary immune response in grandparents re-exposed to chickenpox

**DOI:** 10.1038/s41598-018-28930-9

**Published:** 2018-07-12

**Authors:** B. Ogunjimi, J. Van den Bergh, P. Meysman, S. Heynderickx, K. Bergs, H. Jansens, E. Leuridan, A. Vorsters, H. Goossens, K. Laukens, N. Cools, Viggo Van Tendeloo, N. Hens, P. Van Damme, Evelien Smits, Ph. Beutels

**Affiliations:** 1 Centre for Health Economics Research & Modeling Infectious Diseases (CHERMID), Vaccine & Infectious Disease Institute (VAXINFECTIO), University of Antwerp, Antwerp, Belgium; 2 Interuniversity Institute for Biostatistics and Statistical Bioinformatics, Hasselt University, Hasselt, Belgium; 3 Department of Pediatrics, Antwerp University Hospital, Edegem, Belgium; 4 Laboratory of Experimental Hematology (LEH), Vaccine & Infectious Disease Institute (VAXINFECTIO), University of Antwerp, Antwerp, Belgium; 5 Department of Pediatrics, Ghent University Hospital, Ghent, Belgium; 6 Antwerp Unit for Data Analysis and Computation in Immunology and Sequencing (AUDACIS), Antwerp, Belgium; 7 Advanced Database Research and Modelling (ADReM), Department of Mathematics and Computer Science, University of Antwerp, Antwerp, Belgium; 8 Biomedical Informatics Research Network Antwerp (BIOMINA), University of Antwerp/Antwerp University Hospital, Edegem, Belgium; 9 Center for Cell Therapy and Regenerative Medicine, Antwerp University Hospital, Edegem, Belgium; 10 Department of Laboratory Medicine, Antwerp University Hospital, Edegem, Belgium; 11 Centre for the Evaluation of Vaccination (CEV), Vaccine & Infectious Disease Institute (VAXINFECTIO), University of Antwerp, Antwerp, Belgium; 12 Laboratory of Medical Microbiology, Vaccine & Infectious Disease Institute (VAXINFECTIO), University of Antwerp, Antwerp, Belgium; 13 Center for Oncological Research Antwerp, University of Antwerp, Antwerp, Belgium; 14 School of Public Health and Community Medicine, The University of New South Wales, Sydney, Australia

Correction to: *Scientific Reports* 10.1038/s41598-017-01024-8, published online 24 April 2017

The original version of this Article contained an error in the spelling of H. Jansens, which was incorrectly given as H. Jansen in the Article, and as H. Janssen in the Supplementary Information file.

This has now been corrected in the HTML and PDF versions of this Article, and in the Supplementary Information file.

