# Supplementary material for: Multidisciplinary study of the secondary immune response in grandparents re-exposed to chickenpox
Source: Sci Rep. 2017 Apr 24;7:1077. doi: 10.1038/s41598-017-01024-8 (PMC5430877; doi:10.1038/s41598-017-01024-8)
Supplement: Supplementary file 1 — Supplementary Figures [file 41598_2017_1024_MOESM1_ESM.pdf]

Multidisciplinary study of the secondary immune response in grandparents re-exposed to chickenpox

B Ogunjimi, J Van den Bergh, P Meysman, S Heyndericks, K Bergs, H Jansens, E Leuridan, A Vorsters, H Goossens, K Laukens, N Cools, V Van Tendeloo, N Hens, P Van Damme, E Smits and Ph Beutels

Supplemental Figure 1: T-cell subset proportions in VZV-specific T-cells in re-exposed grandparents.

Note: T-cell subset proportions are shown, using 2.5-97.5 percentiles, up to one year after re-exposure to chickenpox (T0, ≤ 1 week; T1, > 1 week and ≤ 4 weeks; T2, > 4 weeks and ≤ 7 weeks; T3, > 7 weeks and ≤ 20 weeks; T4, > 20 weeks and ≤ 39 weeks; T5, > 39 weeks).

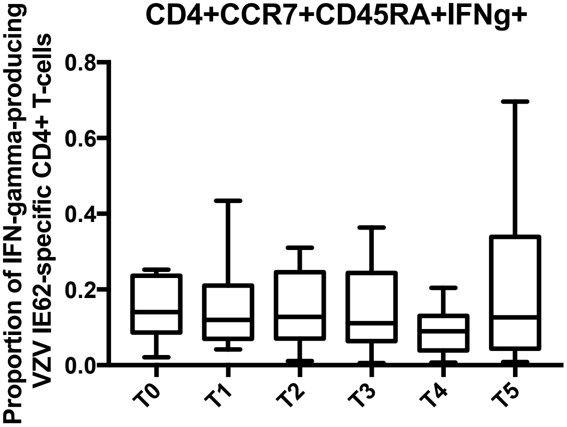
CD4+CCR7+CD45RA+IFNg+
Proportion of IFN-gamma-producing VZV IE62-specific CD4+ T-cells
0.0
0.2
0.4
0.6
0.8
T0
T1
T2
T3
T4
T5

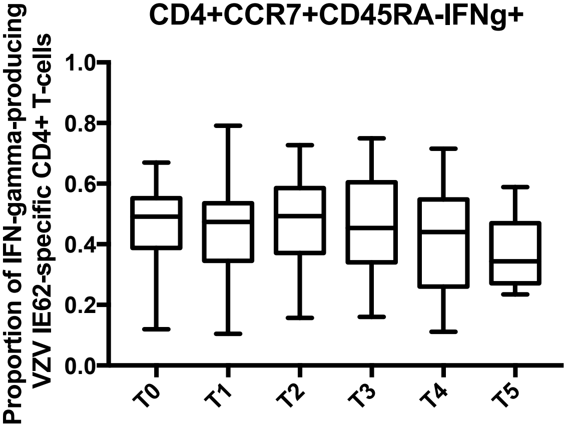
CD4+CCR7+CD45RA-IFNg+
Proportion of IFN-gamma-producing VZV IE62-specific CD4+ T-cells
0.0
0.2
0.4
0.6
0.8
1.0
T0
T1
T2
T3
T4
T5

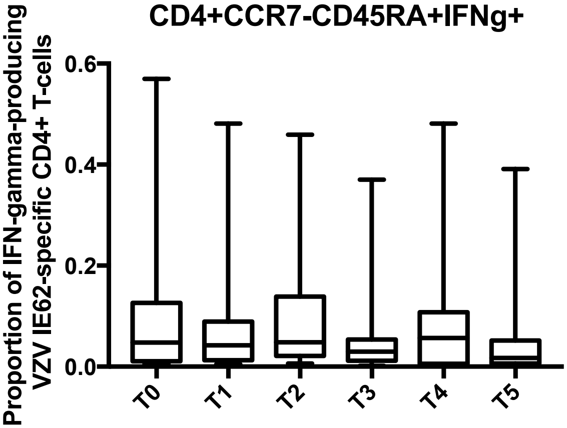
CD4+CCR7-CD45RA+IFNg+
Proportion of IFN-gamma-producing VZV IE62-specific CD4+ T-cells
0.0
0.2
0.4
0.6
T0
T1
T2
T3
T4
T5

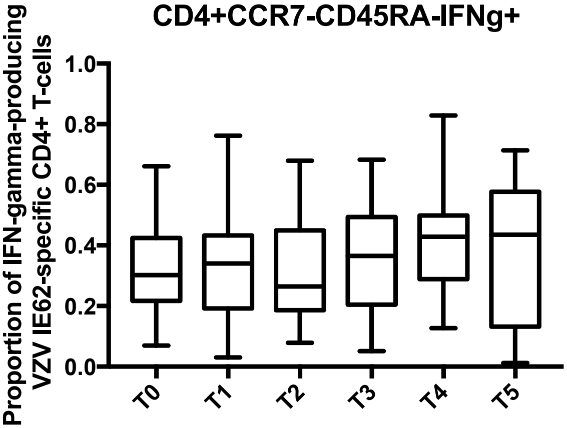
CD4+CCR7-CD45RA-IFNg+
Proportion of IFN-gamma-producing VZV IE62-specific CD4+ T-cells
0.0
0.2
0.4
0.6
0.8
1.0
T0
T1
T2
T3
T4
T5

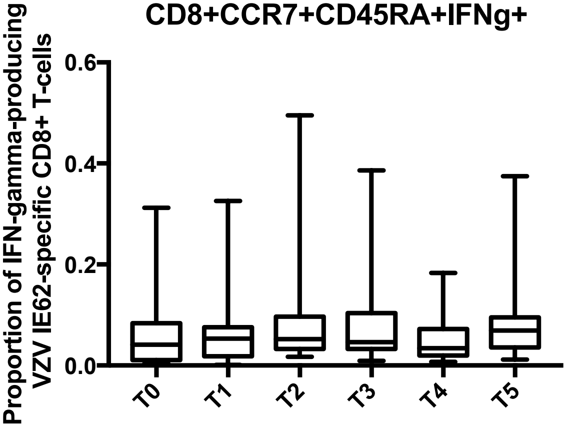
CD8+CCR7+CD45RA+IFNg+
Proportion of IFN-gamma-producing VZV IE62-specific CD8+ T-cells
0.6
0.4
0.2
0.0
T0
T1
T2
T3
T4
T5

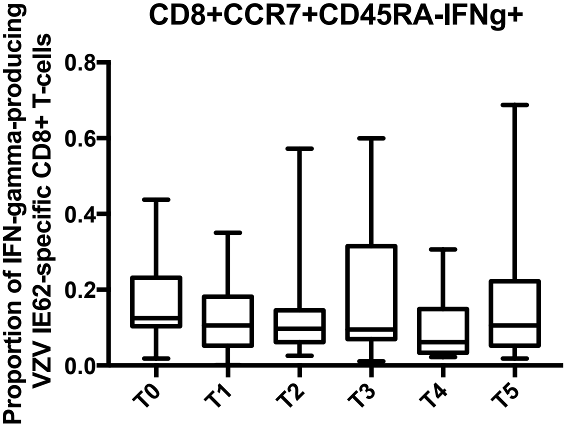
CD8+CCR7+CD45RA-IFNg+
Proportion of IFN-gamma-producing VZV IE62-specific CD8+ T-cells
0.8
0.6
0.4
0.2
0.0
T0
T1
T2
T3
T4
T5

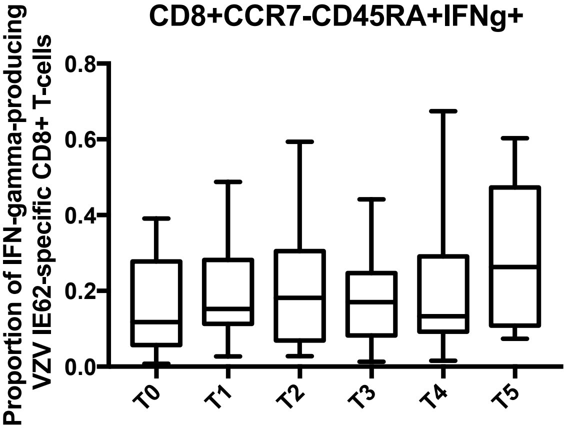
CD8+CCR7-CD45RA+IFNg+
Proportion of IFN-gamma-producing VZV IE62-specific CD8+ T-cells
0.8
0.6
0.4
0.2
0.0
T0
T1
T2
T3
T4
T5

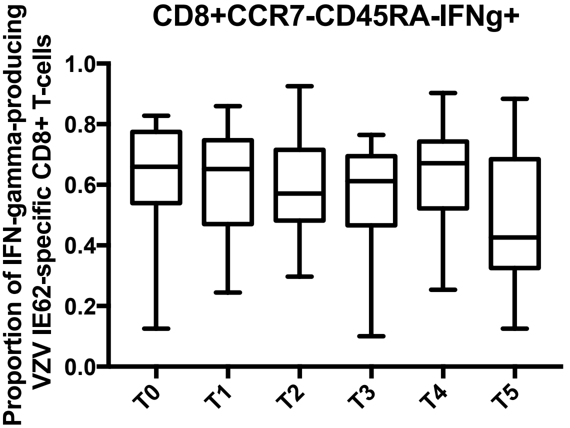
CD8+CCR7-CD45RA-IFNg+
Proportion of IFN-gamma-producing VZV IE62-specific CD8+ T-cells
1.0
0.8
0.6
0.4
0.2
0.0
T0
T1
T2
T3
T4
T5

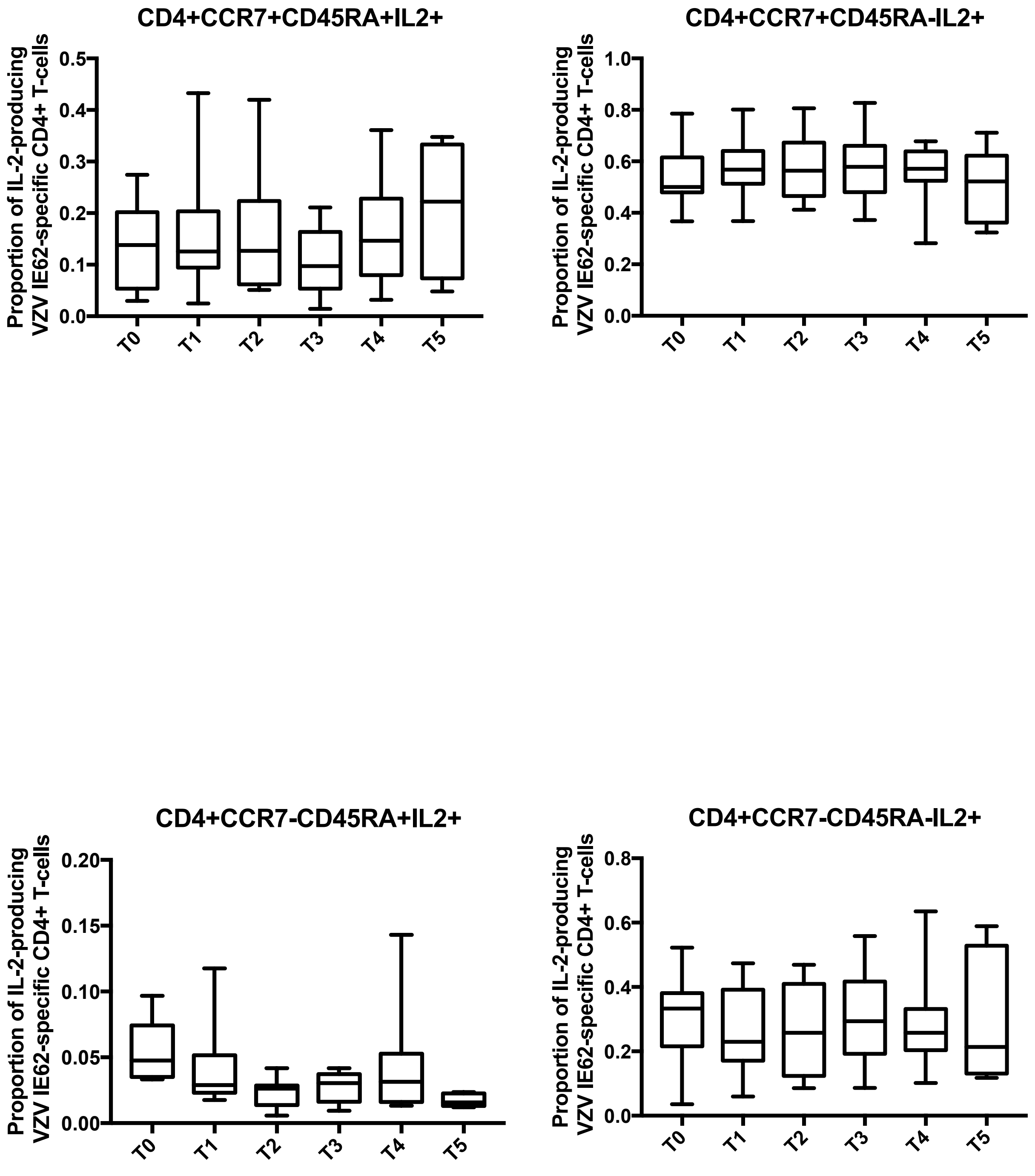

CD4+CCR7+CD45RA+IL2+
CD4+CCR7+CD45RA-IL2+
CD4+CCR7-CD45RA+IL2+
CD4+CCR7-CD45RA-IL2+
Proportion of IL-2-producing VZV IE62-specific CD4+ T-cells
T0
T1
T2
T3
T4
T5

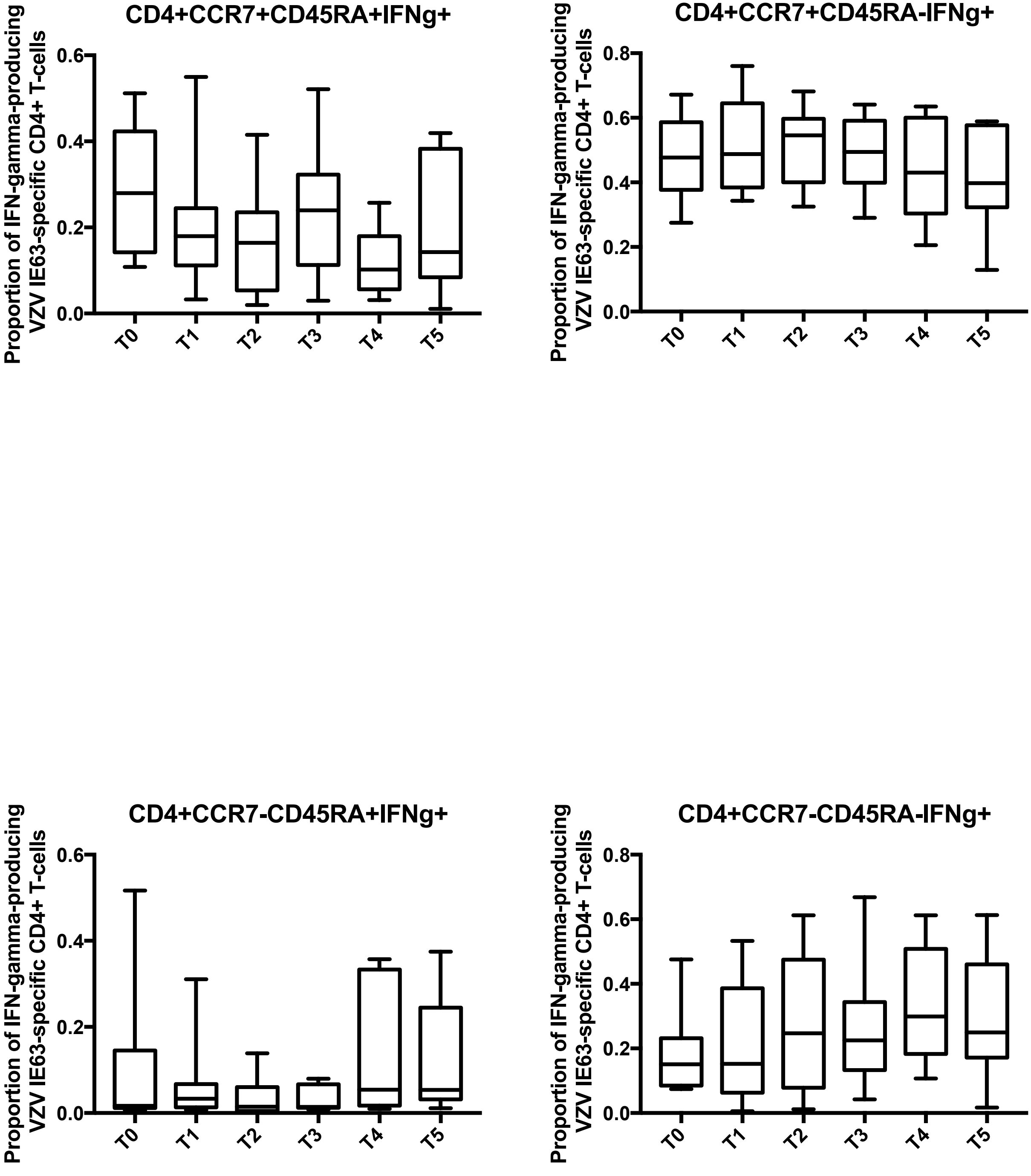
CD4+CCR7+CD45RA+IFNg+
CD4+CCR7+CD45RA-IFNg+
CD4+CCR7-CD45RA+IFNg+
CD4+CCR7-CD45RA-IFNg+
Proportion of IFN-gamma-producing VZV IE63-specific CD4+ T-cells
0.0
0.2
0.4
0.6
0.8
T0
T1
T2
T3
T4
T5

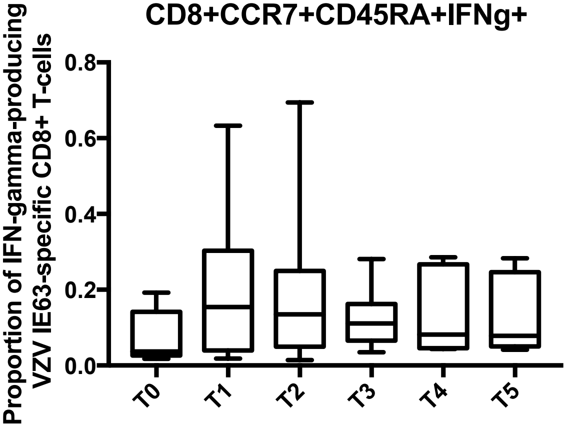
CD8+CCR7+CD45RA+IFNg+
Proportion of IFN-gamma-producing VZV IE63-specific CD8+ T-cells
0.0
0.2
0.4
0.6
0.8
T0
T1
T2
T3
T4
T5

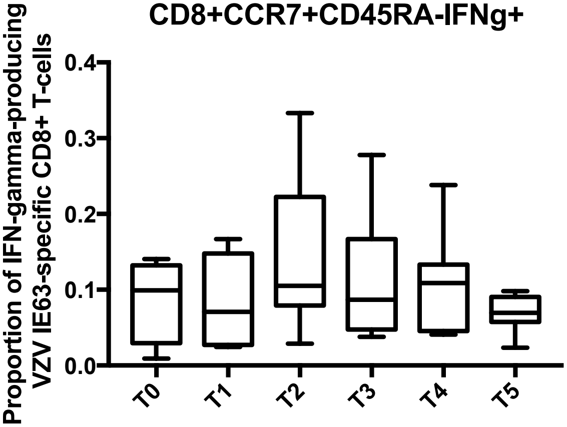
CD8+CCR7+CD45RA-IFNg+
Proportion of IFN-gamma-producing VZV IE63-specific CD8+ T-cells
0.0
0.1
0.2
0.3
0.4
T0
T1
T2
T3
T4
T5

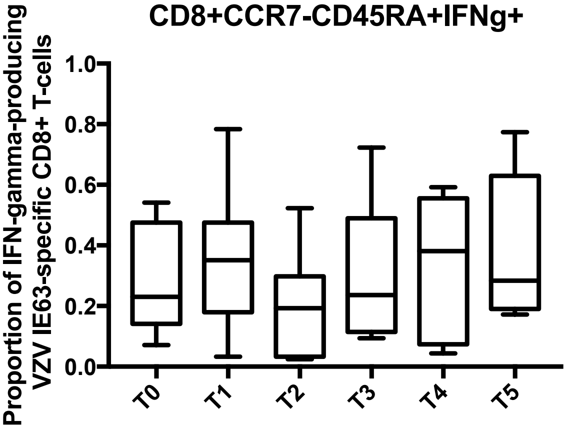
CD8+CCR7-CD45RA+IFNg+
Proportion of IFN-gamma-producing VZV IE63-specific CD8+ T-cells
0.0
0.2
0.4
0.6
0.8
1.0
T0
T1
T2
T3
T4
T5

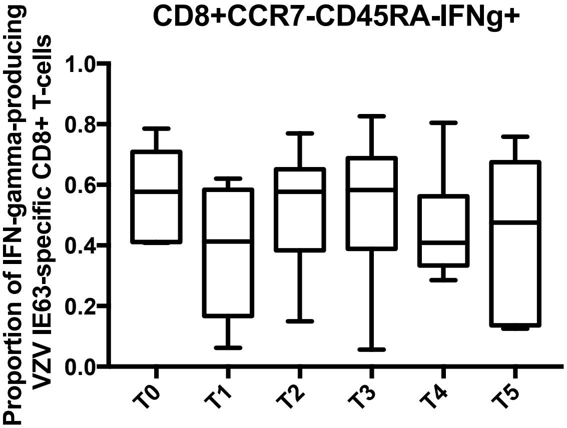
CD8+CCR7-CD45RA-IFNg+
Proportion of IFN-gamma-producing VZV IE63-specific CD8+ T-cells
0.0
0.2
0.4
0.6
0.8
1.0
T0
T1
T2
T3
T4
T5

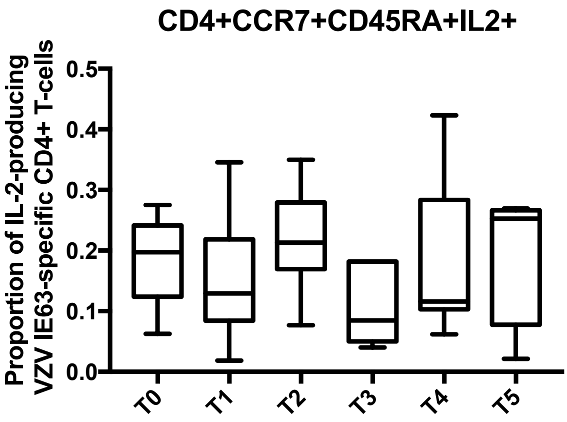
CD4+CCR7+CD45RA+IL2+
Proportion of IL-2-producing VZV IE63-specific CD4+ T-cells
0.0
0.1
0.2
0.3
0.4
0.5
T0
T1
T2
T3
T4
T5

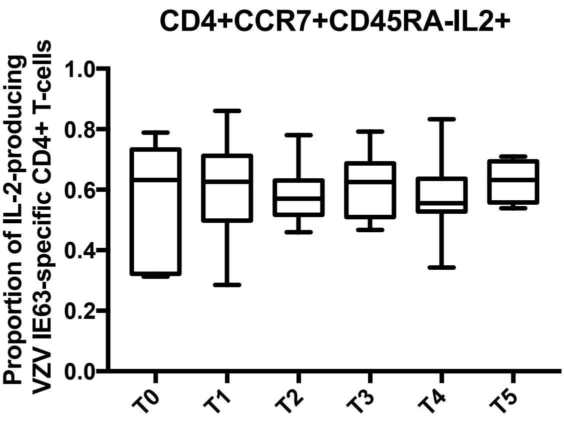
CD4+CCR7+CD45RA-IL2+
Proportion of IL-2-producing VZV IE63-specific CD4+ T-cells
0.0
0.2
0.4
0.6
0.8
1.0
T0
T1
T2
T3
T4
T5

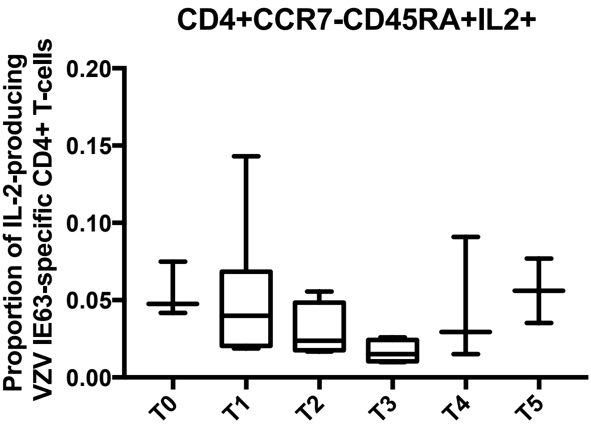
CD4+CCR7-CD45RA+IL2+
Proportion of IL-2-producing VZV IE63-specific CD4+ T-cells
0.00
0.05
0.10
0.15
0.20
T0
T1
T2
T3
T4
T5

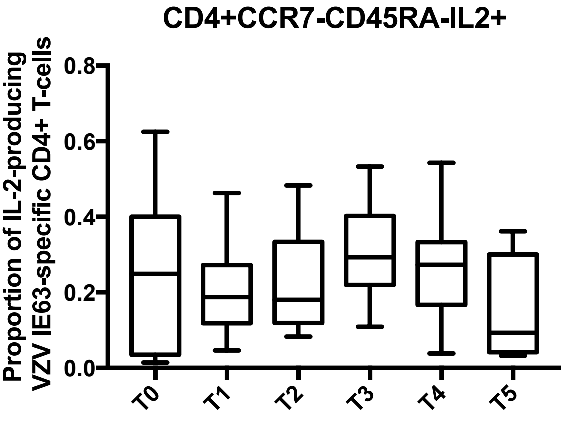
CD4+CCR7-CD45RA-IL2+
Proportion of IL-2-producing VZV IE63-specific CD4+ T-cells
0.0
0.2
0.4
0.6
0.8
T0
T1
T2
T3
T4
T5

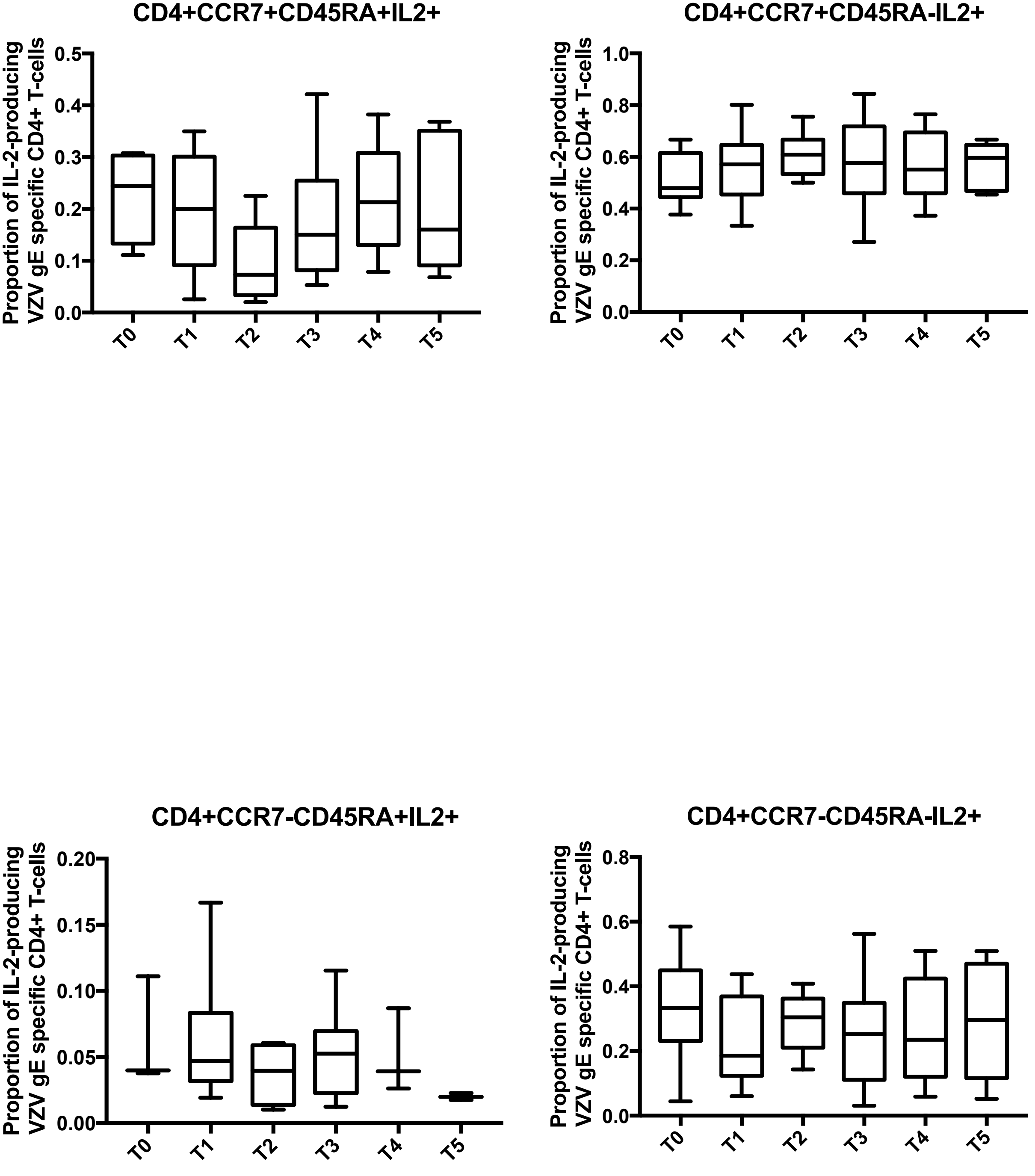
CD4+CCR7+CD45RA+IL2+
Proportion of IL-2-producing VZV gE specific CD4+ T-cells
0.0
0.1
0.2
0.3
0.4
0.5
T0
T1
T2
T3
T4
T5
CD4+CCR7+CD45RA-IL2+
Proportion of IL-2-producing VZV gE specific CD4+ T-cells
0.0
0.2
0.4
0.6
0.8
1.0
T0
T1
T2
T3
T4
T5
CD4+CCR7-CD45RA+IL2+
Proportion of IL-2-producing VZV gE specific CD4+ T-cells
0.00
0.05
0.10
0.15
0.20
T0
T1
T2
T3
T4
T5
CD4+CCR7-CD45RA-IL2+
Proportion of IL-2-producing VZV gE specific CD4+ T-cells
0.0
0.2
0.4
0.6
0.8
T0
T1
T2
T3
T4
T5

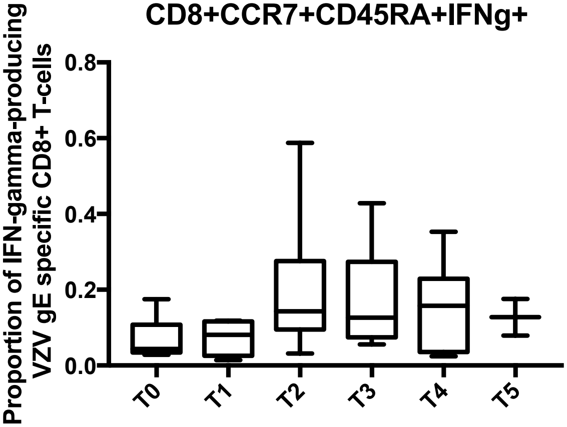
CD8+CCR7+CD45RA+IFNg+
Proportion of IFN-gamma-producing VZV gE specific CD8+ T-cells
0.0
0.2
0.4
0.6
0.8
T0
T1
T2
T3
T4
T5

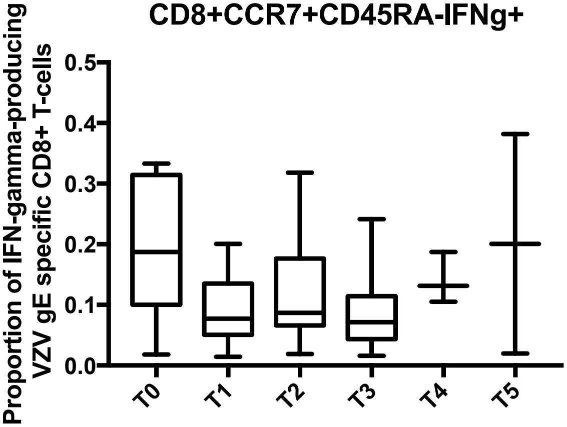
CD8+CCR7+CD45RA-IFNg+
Proportion of IFN-gamma-producing VZV gE specific CD8+ T-cells
0.0
0.1
0.2
0.3
0.4
0.5
T0
T1
T2
T3
T4
T5

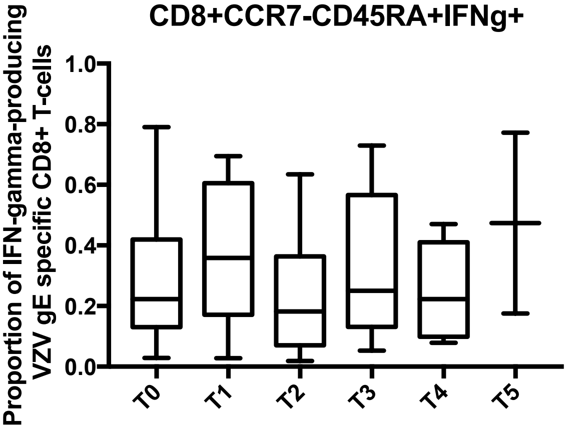
CD8+CCR7-CD45RA+IFNg+
Proportion of IFN-gamma-producing VZV gE specific CD8+ T-cells
0.0
0.2
0.4
0.6
0.8
1.0
T0
T1
T2
T3
T4
T5

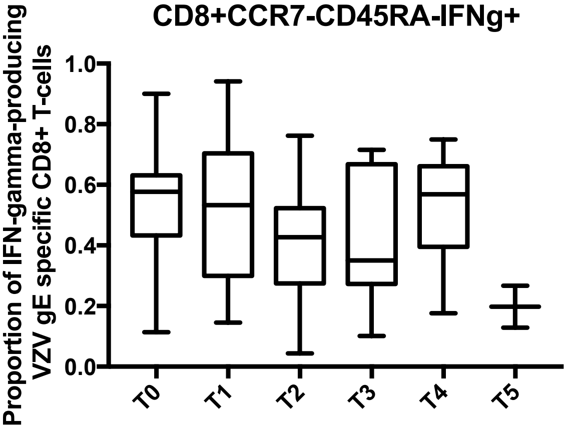
CD8+CCR7-CD45RA-IFNg+
Proportion of IFN-gamma-producing VZV gE specific CD8+ T-cells
0.0
0.2
0.4
0.6
0.8
1.0
T0
T1
T2
T3
T4
T5

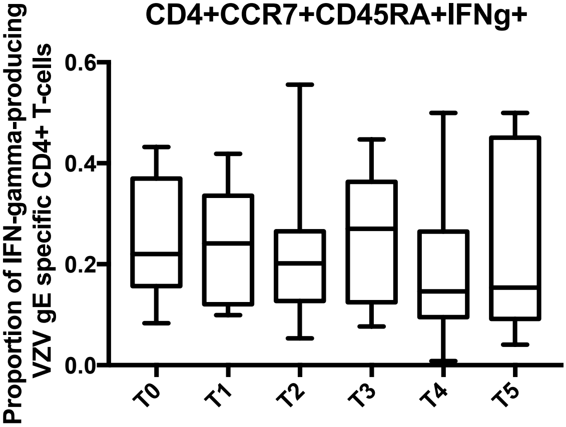
CD4+CCR7+CD45RA+IFNg+
Proportion of IFN-gamma-producing
VZV gE specific CD4+ T-cells
0.0
0.2
0.4
0.6
T0
T1
T2
T3
T4
T5

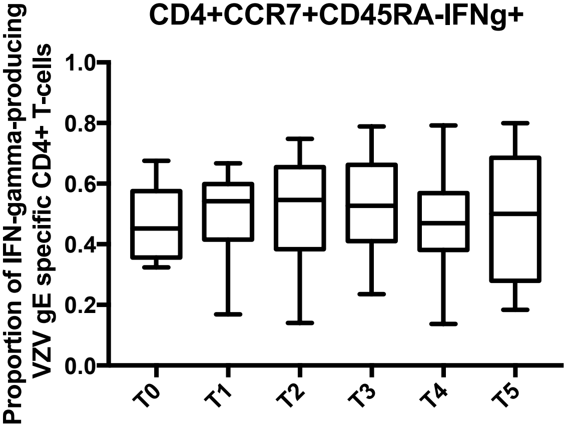
CD4+CCR7+CD45RA-IFNg+
Proportion of IFN-gamma-producing
VZV gE specific CD4+ T-cells
0.0
0.2
0.4
0.6
0.8
1.0
T0
T1
T2
T3
T4
T5

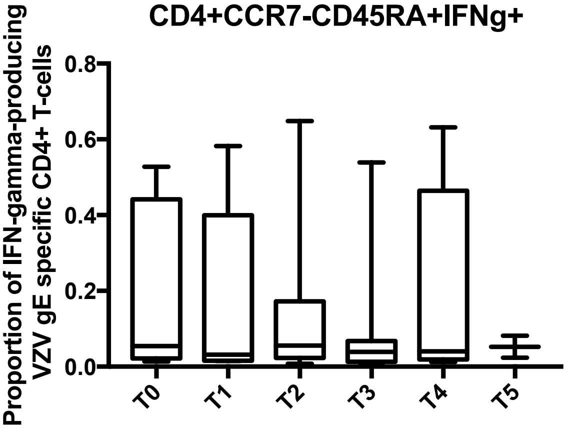
CD4+CCR7-CD45RA+IFNg+
Proportion of IFN-gamma-producing
VZV gE specific CD4+ T-cells
0.0
0.2
0.4
0.6
0.8
T0
T1
T2
T3
T4
T5

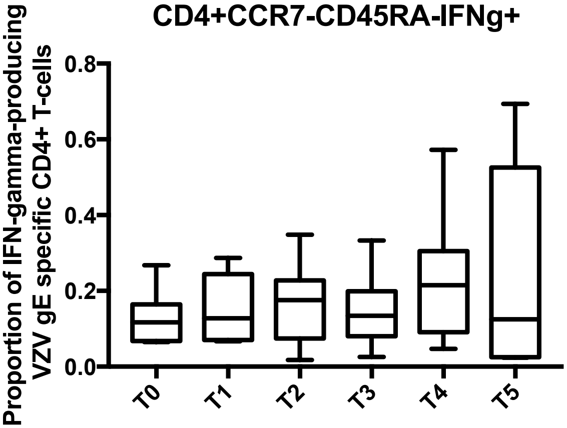
CD4+CCR7-CD45RA-IFNg+
Proportion of IFN-gamma-producing
VZV gE specific CD4+ T-cells
0.0
0.2
0.4
0.6
0.8
T0
T1
T2
T3
T4
T5

Supplemental Figure 2: VZV-specific CD8+ TCRb read frequency for three re-exposed grandparents on three different time points after re-exposure.

Note: scatter plot shows data on logarithmic scale ("0" results were replaced by 0.00002 for visualisation purposes).

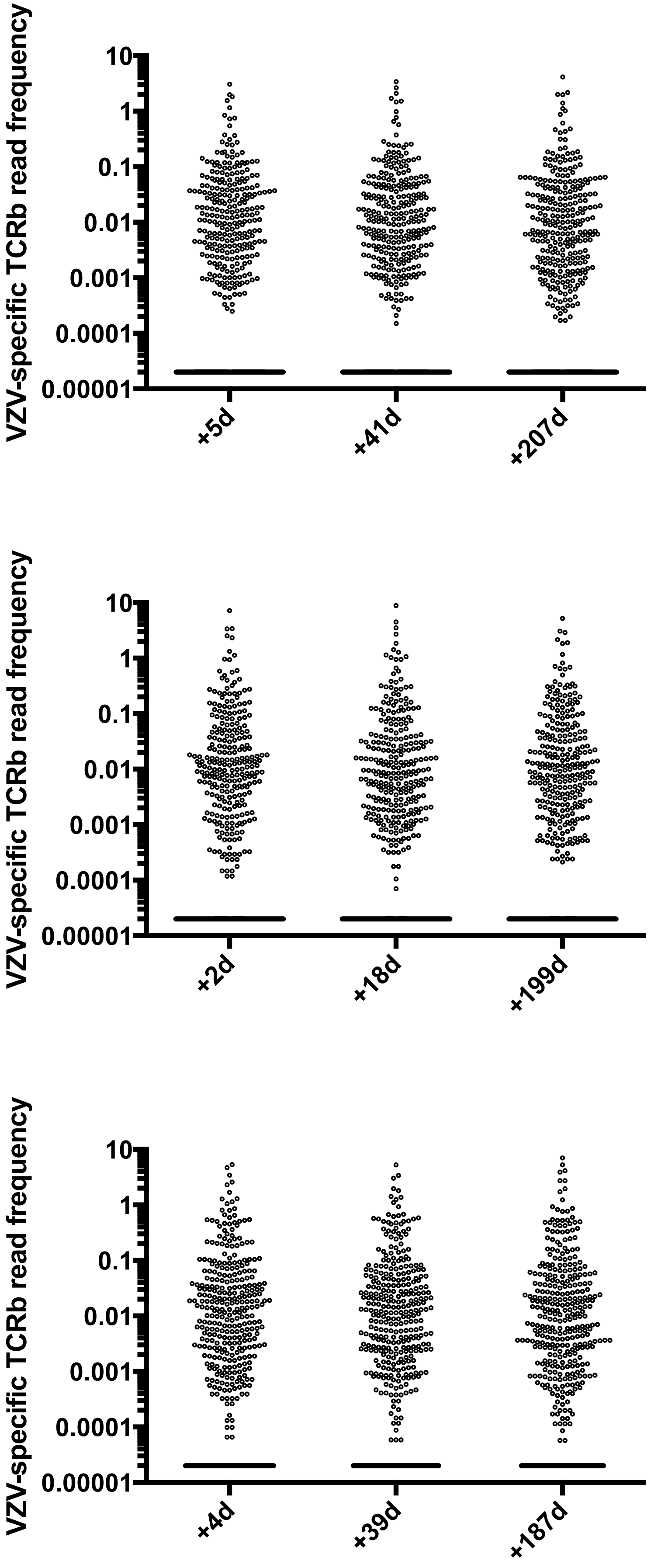
VZV-specific TCRb read frequency
10
1
0.1
0.01
0.001
0.0001
0.00001
+5d
+41d
+207d
+2d
+18d
+199d
+4d
+39d
+187d

Supplemental Figure 3: Individual fits of observed vs. predicted VZV-antibody titers for re-exposed grandparents

Note: On the x-axis time is shown in days since re-exposure to chickenpox and on the y-axis VZV-antibody titer is shown in mIU/ml.

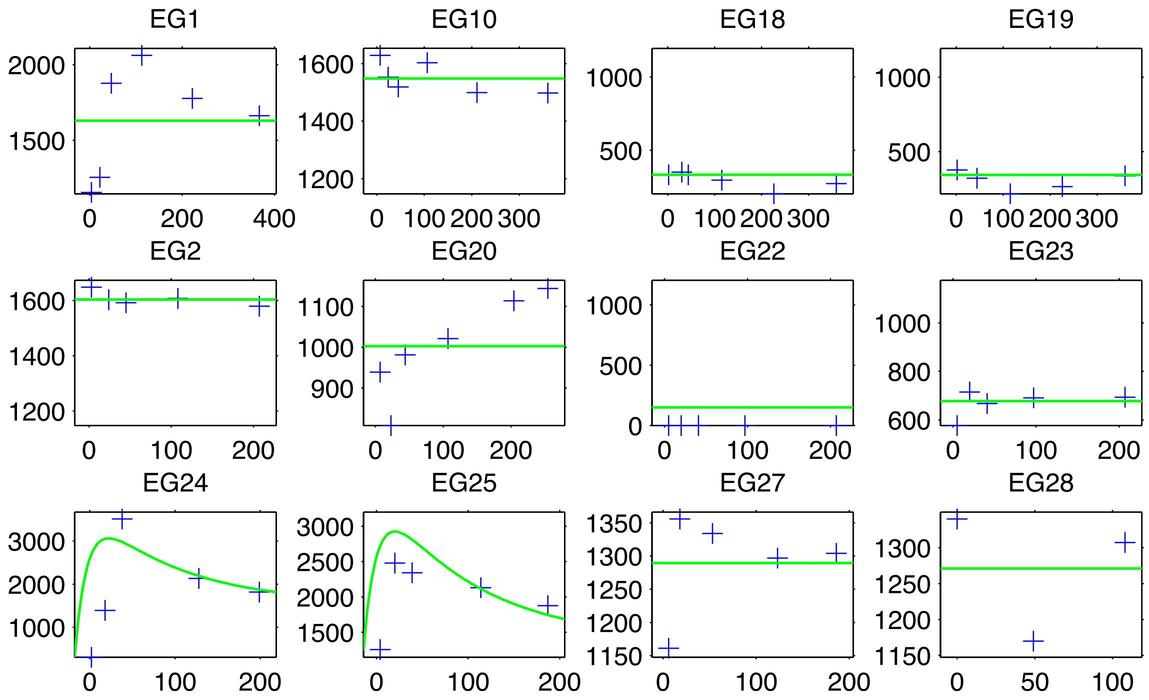
EG1
2000
1500
0
200
400
EG10
1600
1400
1200
0
100
200
300
EG18
1000
500
0
100
200
300
EG19
1000
500
0
100
200
300
EG2
1600
1400
1200
0
100
200
EG20
1100
1000
900
0
100
200
EG22
1000
500
0
0
100
200
EG23
1000
800
600
0
100
200
EG24
3000
2000
1000
0
100
200
EG25
3000
2500
2000
1500
0
100
200
EG27
1350
1300
1250
1200
1150
0
100
200
EG28
1300
1250
1200
1150
0
50
100

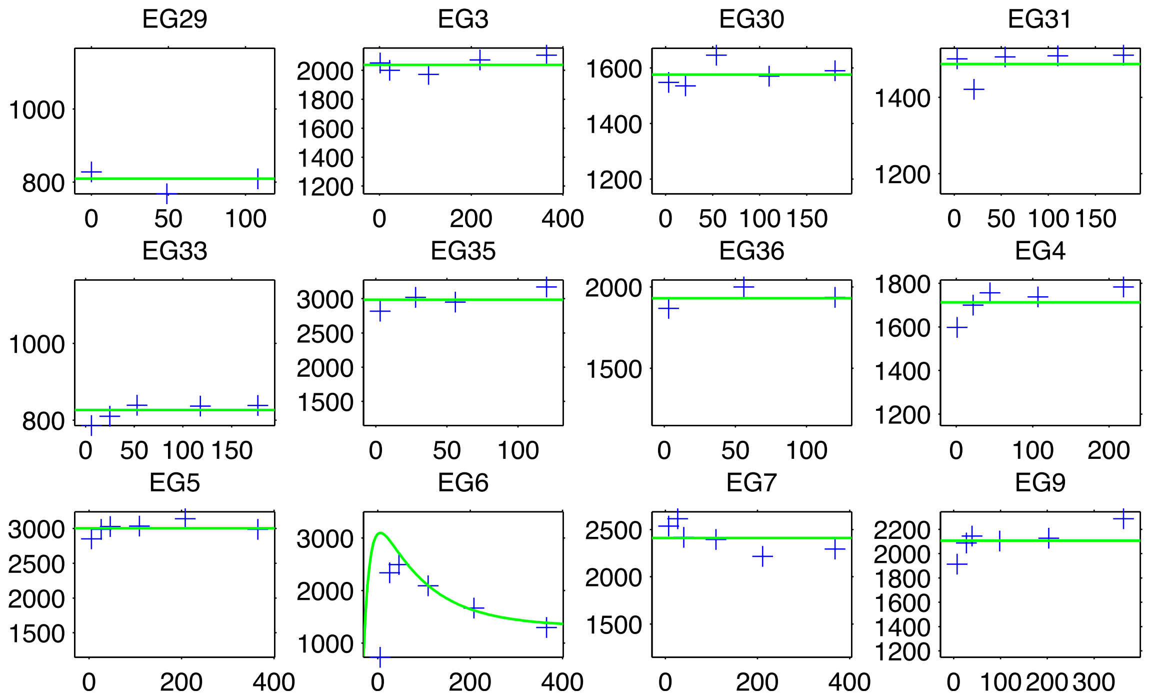
EG29
EG3
EG30
EG31
EG33
EG35
EG36
EG4
EG5
EG6
EG7
EG9

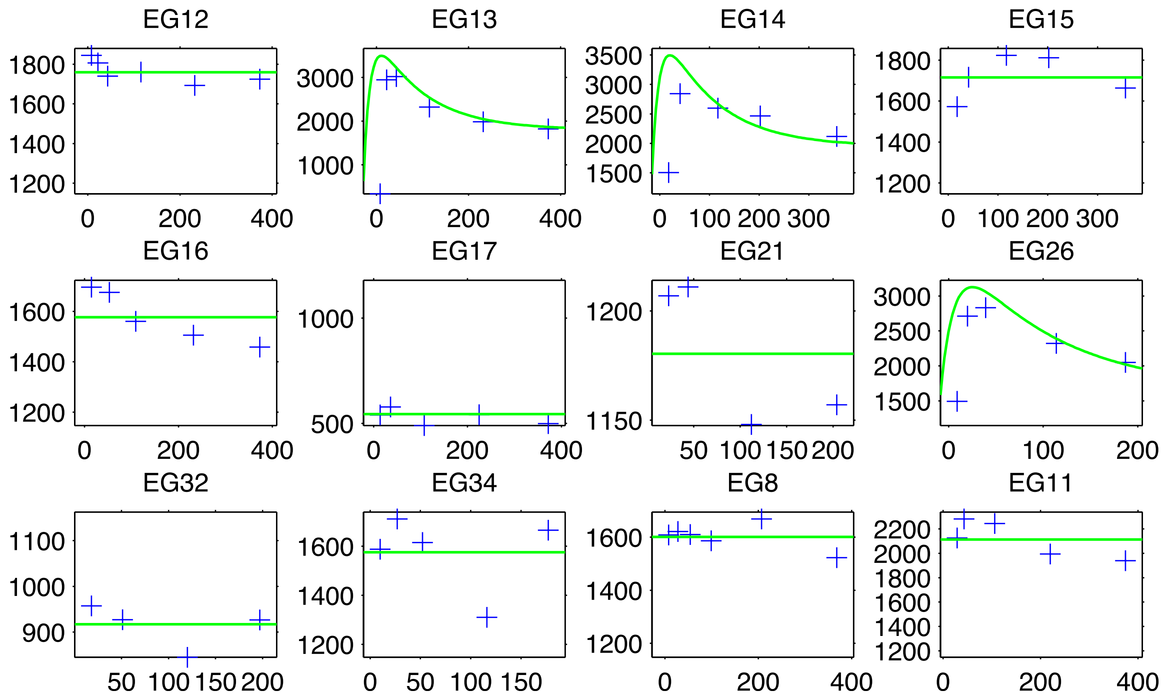
EG12
EG13
EG14
EG15
EG16
EG17
EG21
EG26
EG32
EG34
EG8
EG11

Supplemental Figure 4: Individual fits of observed vs. predicted VZV IE62 specific CD4+ IL-2-producing T-cells for re-exposed grandparents

Note: On the x-axis time is shown in days since re-exposure to chickenpox and on the y-axis the number of CD4+ IL-2-producing T-cells per 100,000 CD4+ T-cells are shown.

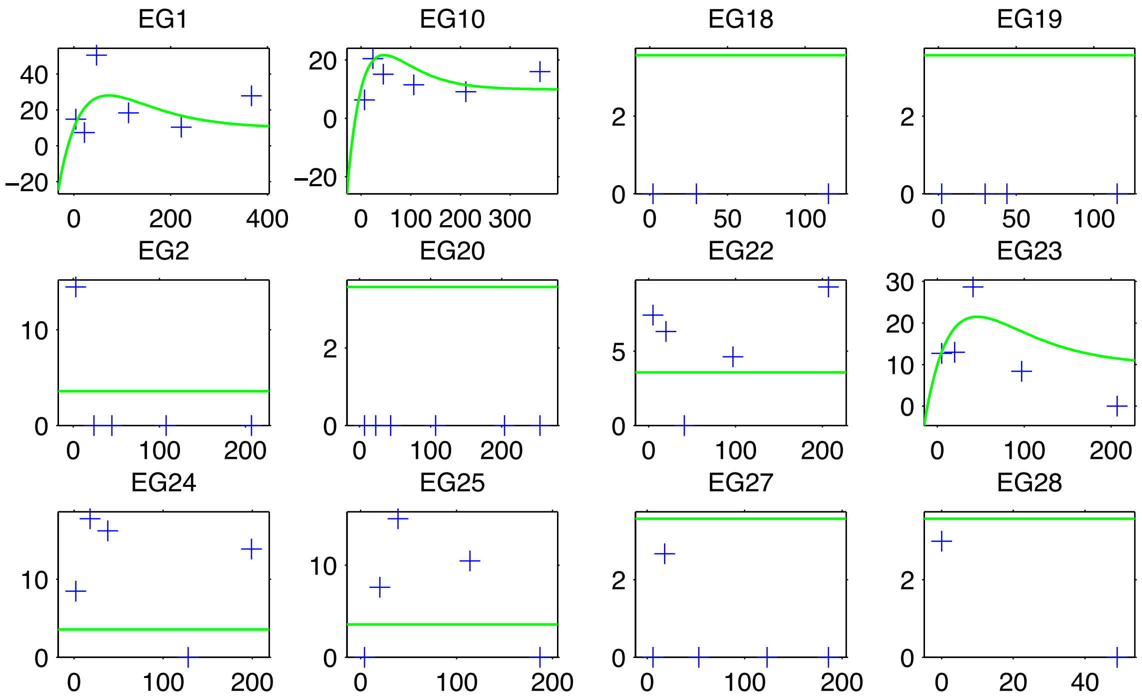
EG1
EG10
EG18
EG19
EG2
EG20
EG22
EG23
EG24
EG25
EG27
EG28

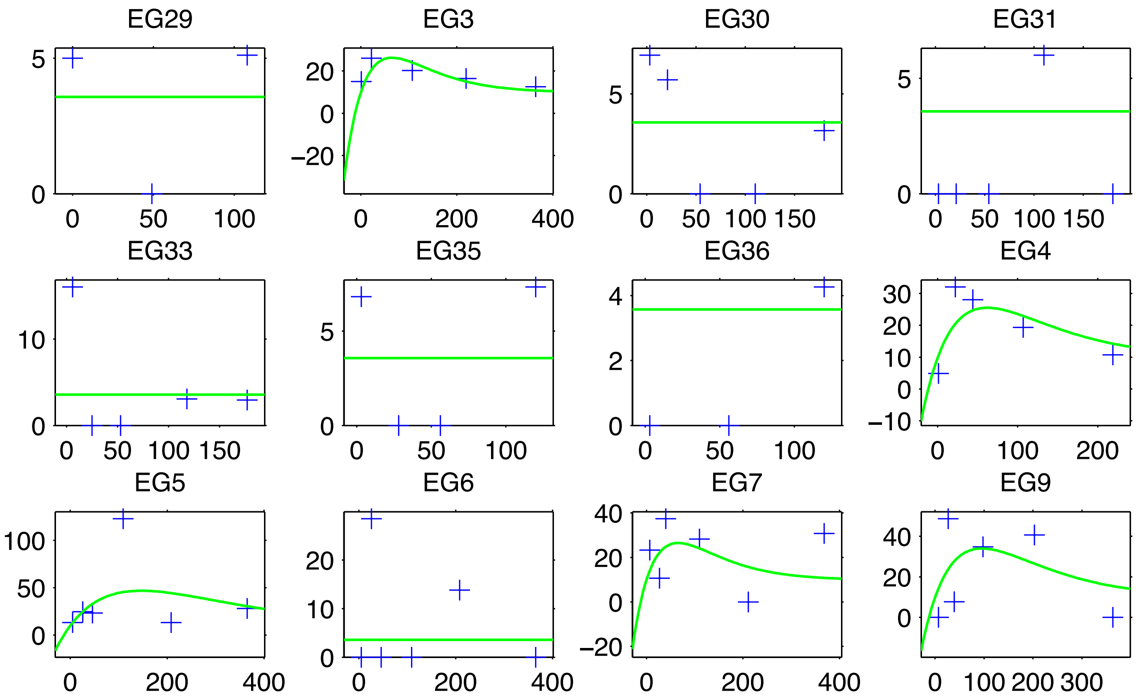
EG29
EG3
EG30
EG31
EG33
EG35
EG36
EG4
EG5
EG6
EG7
EG9

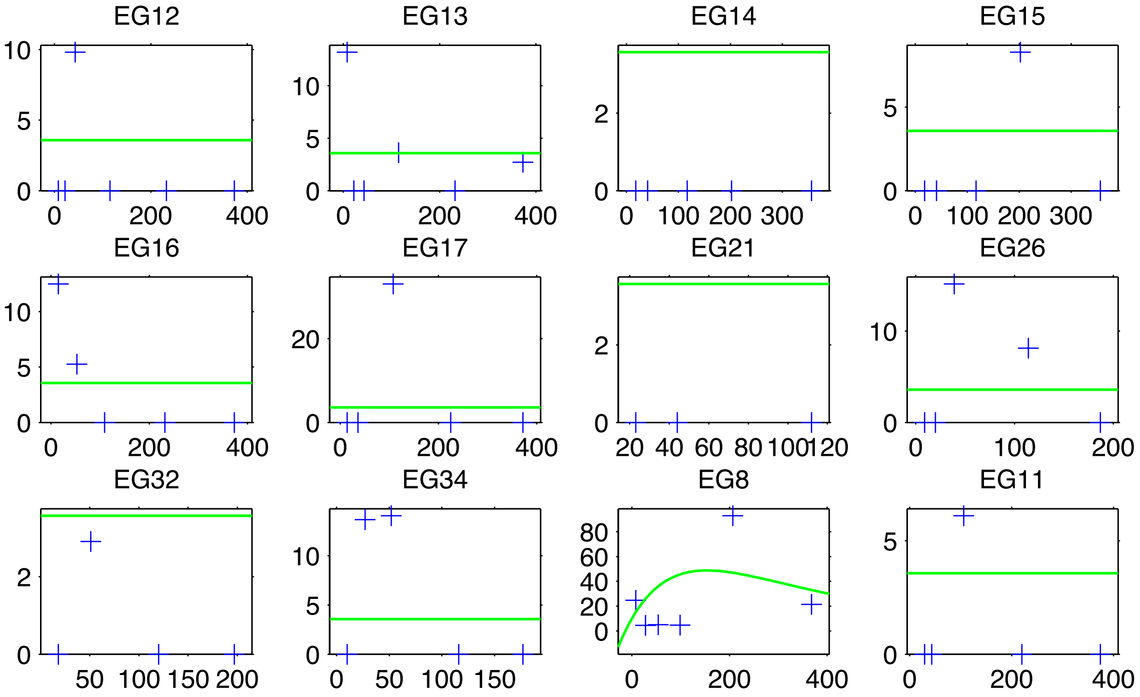
EG12
EG13
EG14
EG15
EG16
EG17
EG21
EG26
EG32
EG34
EG8
EG11
